# Win–Win More Sustainable Routes for Acetic Acid Synthesis

**DOI:** 10.1021/acssuschemeng.4c07324

**Published:** 2025-01-22

**Authors:** Juan D. Medrano-García, Raul Calvo-Serrano, Haining Tian, Gonzalo Guillén-Gosálbez

**Affiliations:** † Institute for Chemical and Bioengineering, Department of Chemistry and Applied Biosciences, ETH Zurich, Vladimir Prelog Weg 1, 8093 Zurich, Switzerland; ‡ Departament d’Enginyeria Química i Ciència de Materials, Institut Quimic de Sarrià, Universitat Ramon Llull, Via Augusta 390, 08017 Barcelona, Spain; § Department of Chemistry—Ångström Laboratory, Physical Chemistry, Uppsala University, Box 521, 75120Uppsala, Sweden

**Keywords:** green acetic acid, green carbon monoxide, biogas, semiartificial photosynthesis (SAP), life-cycle assessment (LCA), process simulation, win−win scenario

## Abstract

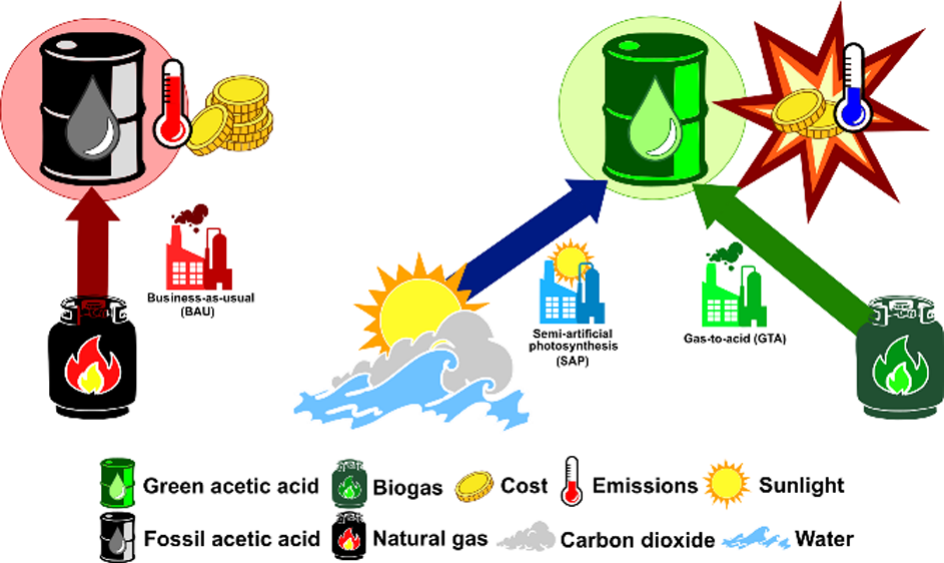

Current efforts to decarbonize the chemical sector by using captured CO_2_ and electrolytic H_2_ typically lead to high production costs and environmental collateral damage. Hence, there is a clear need to look for alternative, more efficient synthesis routes that could pave the way for a fully sustainable chemical industry. Bearing this in mind, here, we evaluate the economic and environmental implications of two low technology readiness level (TRL) novel single-step synthesis routes for acetic acid production using CO_2_ as a raw material: gas-to-acid methane carboxylation and semiartificial photosynthesis. Using process simulation and life-cycle assessment, we determine that these pathways, under a specific set of assumptions, could outperform the business-as-usual methanol carbonylation process at their current development state in terms of global warming, human health, ecosystem quality, and resource scarcity impacts, showing no signs of burden shifting. Furthermore, these routes also result in lower production costs derived from the reduced energy requirement associated with a single synthesis step. Overall, our preliminary results of the low TRL technologies based on experimental data highlight the potential economic and environmental benefits of exploring alternative synthesis routes, which could help bridge the current fossil-based industrial landscape to a more sustainable future.

## Abstract 2

Direct acetic acid synthesis routes from CO_2_ can lead to an environmental and economic win−win scenario compared with the business-as-usual pathway.

## Introduction

The chemical sector is responsible for generating about 5% of the global CO_2_ emissions worldwide.^1^ For this reason, direct action is needed to reduce the environmental impact associated with chemical production pathways. Usually, these efforts focus on the substitution of current fossil-based inputs with renewable energy and feedstocks, essentially turning the subsequent downstream processes “green” in addition to minimizing the changes in the current infrastructure. The most prominent example of this practice is the use of direct air-captured (DAC) CO_2_ and/or electrolytic green H_2_ powered by wind or solar energy in the synthesis of methanol,^2^ ammonia,^3^ or Fischer–Tropsch electrofuels.^4^ However, despite the clear advantage in terms of curbing carbon emissions, these changes usually lead to environmental burden shifting (i.e., one impact improves at the expense of worsening others) and high production costs.^5^ Given this situation, alternative synthesis routes could play a key role in the transition toward a more sustainable chemical industry, potentially reshaping the supply chains by partially substituting important raw materials and/or completely avoiding full reaction steps and, therefore, reducing energy and feedstock demand due to more efficient reaction pathways. One example of this practice is the use of ethane in the one-step synthesis of the vinyl chloride monomer (VCM), which removes the need for the high-cost and carbon-intensive two-step ethylene balanced process, leading to potential environmental and economic win–win scenarios when evaluated under a more decarbonized future chemical industry prospectively.^6^

Acetic acid is an important chemical used mainly as a precursor for polymers derived from vinyl and cellulose acetate, such as poly(vinyl alcohol) (PVA) or acetate fibers.^7^ Its global demand in 2022 was 17 Mt, consuming approximately 10% of the global methanol production,^8^ and it is expected to grow 25% by 2030.^9^ Currently, acetic acid is mostly produced through the carbonylation of methanol (i.e., methanol reaction with CO), which can be derived from synthesis gas obtained from high-temperature steam methane reforming (SMR) or partial oxidation (POX) of natural gas.^7^ Furthermore, this technology uses a homogeneous catalyst and generates small quantities of propionic acid as a byproduct, which increases the required separation steps and, thus, the energy demand.^10^ As such, the process exhibits high costs and carbon emissions.^11^

Given these limitations, promising alternative and more sustainable routes for acetic acid synthesis have been investigated. Otto et al.^12^ identified 123 CO_2_ utilization reactions with high economic and environmental potential, which include acetic acid direct synthesis from methane and CO_2_. Martín-Espejo et al.^13^ compared the traditional fossil-based synthesis with two biogas-based strategies: an indirect one using dry methane reforming (DMR) and a direct route based on methane carboxylation (i.e., the reaction promoted by Otto et al.). Their results highlight the potential of the direct route, labeled as a ground-breaking and atom-efficient synthesis pathway, while stressing the need for further catalytic progress for its potential implementation. Along these lines, Shavi et al.^14^ reported an 8% methane direct conversion to acetic acid with an outstanding 100% selectivity using a CeO_2_–ZnO supported montmorillonite catalyst at 2 bar and 300 °C.

The direct methane carboxylation route can produce the desired product in a single step with low energy consumption and using readily available raw materials like CO_2_. By extension, other routes capable of selectively synthesizing acetic acid in a single step at mild conditions could also hold great potential. This is the case of photosynthetic acetic acid produced from CO_2_ by some anaerobic bacteria, such as *Moorella thermoacetica*, able to operate optimally at around 60 °C and atmospheric pressure using sunlight as the only energy source.^15^ However, natural photosynthesis, despite displaying great selectivities toward the desired products, usually presents low efficiencies, while the opposite behavior is shown by artificial photosynthesis.^16^ For this reason, semiartificial photosynthesis (SAP) was born.^17^ These hybrid systems synergize by combining artificial photosensitizers that efficiently interact with sunlight and natural catalytic centers that feed from the electrons collected by the photosensitizer and a sacrificial hydrogen donor, such as cysteine, some amines, or water.^18^ For example, Wang and co-workers^19^ studied a hybrid system using perylene diimide derivative and poly(fluorene-*co*-phenylene) with *Moorella thermoacetica* for the production of acetic acid from CO_2_ and cysteine, reporting an activity increase of 300% compared to the natural photosynthesis control system. However, despite the clear potential of these SAP routes, no economic or environmental analysis has been carried out in the literature.

Here, we study the economic and environmental performance of methane carboxylation and SAP processes, comparing them with the fossil, green, and biogas-based conventional synthesis. Our results confirm that these routes have the potential to outperform the business-as-usual (BAU) pathway, both economically and environmentally, under specific feedstock scenarios, setting the path toward the future of acetic acid synthesis and, beyond that, a more sustainable chemical industry.

## Methodology

We evaluated four synthesis routes for acetic acid production. These routes are business-as-usual (BAU) methanol carbonylation, gas-to-acid (GTA) methane carboxylation, and SAP using (1) cysteine and (2) water as hydrogen donors. We build the life-cycle inventory (LCI) of the BAU route from the energy and material balances reported by Dimian and Kiss.^10^ The LCIs of both the GTA and SAP routes were obtained from the material and energy balances of their respective simulated processes using Aspen HYSYS v12 based on experimental data and following common practices.^20^ With the LCIs (foreground system), we perform the life-cycle impact assessment (LCIA) with Brightway2 v2.4.2,^21^ modeling the background system with Ecoinvent 3.8.^22^ Finally, we calculate the economic performance using the capital and operating costs of the scaled plants.^23^ We first present the scenarios followed by the process descriptions, the life-cycle assessment (LCA) methodology, and finally the economic analysis.

### Case Studies

**Figure 1:**
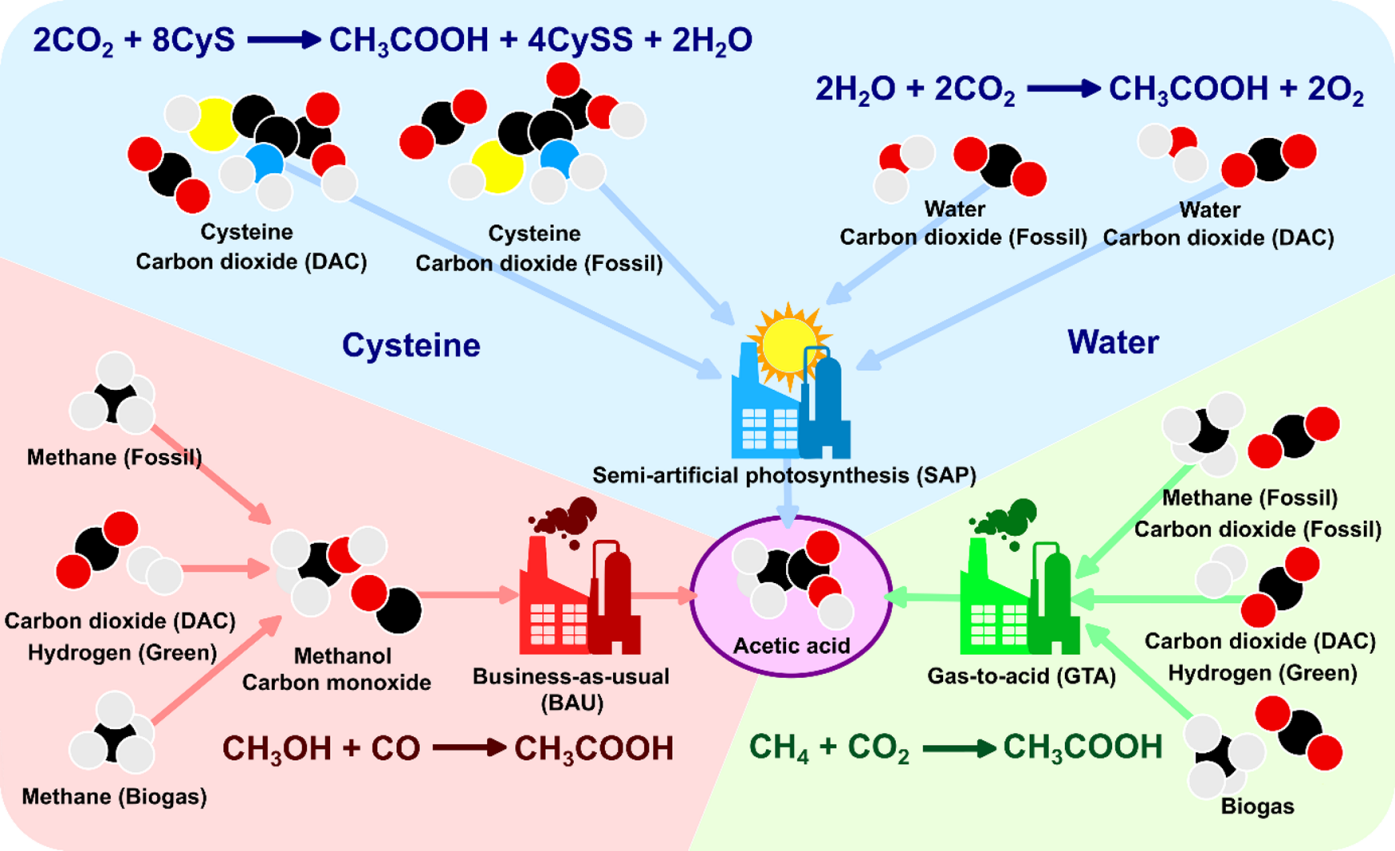
Graphical representation of the assessed scenarios. CyS refers to cysteine (C_3_H_7_NO_2_S), while CySS refers to cystine (C_6_H_12_N_2_O_4_S_2_).

Acetic acid synthesis is evaluated under ten scenarios resulting from the combination of the four process technologies (BAU, GTA, and two SAP options) combined with different raw material sources (Figure 1). The BAU includes a fossil scenario using methanol from natural gas-based SMR and carbon monoxide from methane POX; a green scenario where these two chemicals are synthesized from DAC CO_2_ and wind electrolytic H_2_ (i.e., green methanol from CO_2_ hydrogenation and green CO from reverse water–gas shift (RWGS) of CO_2_ and H_2_); and a biogenic (bio) scenario using biomethane instead of fossil methane as the platform chemical for methanol and CO synthesis. The GTA considers equivalent scenarios (fossil, green, and biogenic) for the usage of the required methane and CO_2_ raw materials. The fossil-GTA scenario considers methane from natural gas and coal power plant-captured CO_2_, while the green-GTA uses DAC CO_2_ and wind electrolytic H_2_ for the synthesis of methane. Finally, bio-GTA uses biogas directly for the synthesis. The SAP route is evaluated from two different configurations. The first one uses CO_2_ as the carbon base with cysteine as the H_2_ donor molecule, while the second one uses water instead. Both of these SAP configurations are evaluated under fossil (coal power plant-captured CO_2_) and green (DAC CO_2_) scenarios.

### Acetic Acid Process Overview

**Figure 2:**
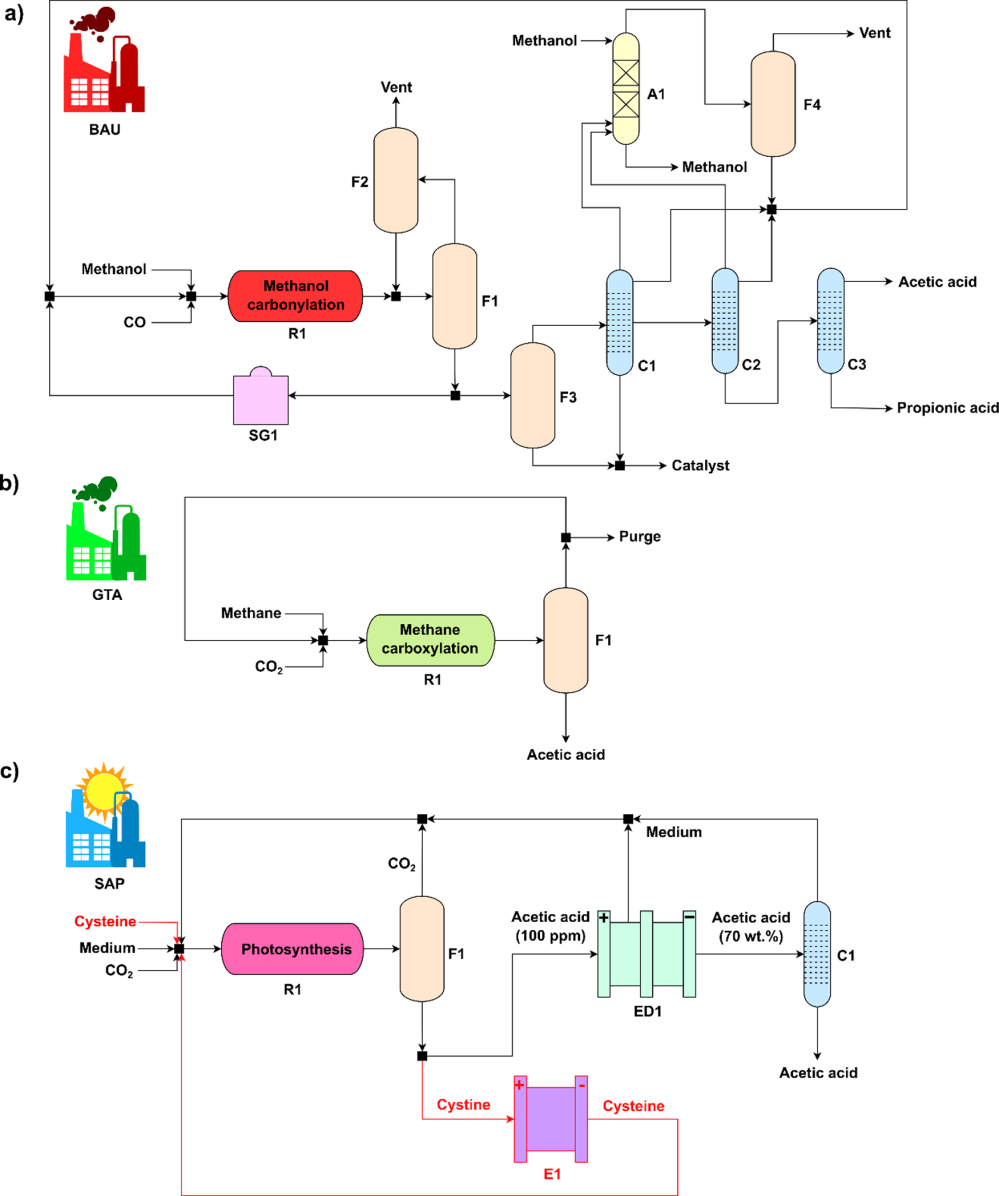
Main acetic acid process flowsheet configurations: (a) methanol carbonylation (BAU); (b) methane carboxylation (GTA); and (c) SAP with (including the red path) and without using cysteine. The codes for the process units are as follows: A, absorbers; C, distillation columns; E, electrolyzers; ED, electrodialysis; F, flash separators; R, reactors; SG, steam generation.

In this section, we describe the three processes used for acetic acid synthesis (Figure 2). More detailed descriptions including process conditions and simulation results can be found in the Supporting Information (Section A).

The BAU methanol carbonylation process (Figure 2a) is based on the Cativa process and uses methanol and CO as raw materials with a homogeneous rhodium catalyst.^10^ The process starts by compressing and heating the raw materials to the reaction conditions before they are fed to the methanol carbonylation reactor (R1). The reactor effluent is cooled and sent to a flash separator (F1). The vapor is further cooled and sent to a second flash (F2), after which the vapor is vented, and the liquid is recycled back to the first flash. The liquid of F1 is split into two streams. The first stream is sent to a steam generation unit (SG1) before going back to the reactor, while the second one is cooled and depressurized before entering a third flash (F3) where most of the catalyst is recovered in the liquid phase. The vapor stream is sent to a three-distillation column train. In the first column (C1), the remaining catalyst is recovered as the bottom product, and impure acetic acid is recovered as a side vapor stream and fed to the next column (C2). In this column, acetic acid is dehydrated, obtaining water as the top product and a mixture of acetic acid and propionic acid, which is sent to the last column (C3), where the two products are obtained as the distillate and bottoms, respectively. Finally, the top vapor streams of both C1 and C2 are sent to the methanol absorption column (A1), where the light ends are recovered and recycled back to the reactor, along with the distillates of C1 and C2.

Moreover, CO and methane syntheses were also simulated in Aspen HYSYS v12. We considered CO to be produced from methane POX followed by cryogenic distillation^24^ for the fossil (natural gas) and bio (biomethane) scenarios, while green CO for the green routes was assumed to be synthesized from RWGS of DAC CO_2_ with wind electrolytic H_2_^4^ followed by pressure swing adsorption.^24^ For methane, we assume the Sabatier process, taking place from electrolytic H_2_ and DAC CO_2_. More details on these process simulations can be found in the Supporting Information (Section A).

The GTA methane carboxylation process (Figure 2b) uses an equimolar flow of methane and CO_2_ to produce acetic acid at 100% selectivity and 8% methane conversion.^14^ After the reaction (R1), the mixture is cooled down to 40 °C. Due to the appreciable difference in volatilities, most of the acetic acid is recovered at high purity (99.99 wt %) in a flash separator (F1) simply by condensation, while the unreacted raw materials and the unseparated acetic acid are recycled back to the reactor after a small purge.

In the SAP process (Figure 2c), CO_2_, the H_2_ donor (i.e., cysteine or water), and the medium makeup are mixed before entering the photosynthesis reactor (R1).^19^ After the reaction, the catalytic system (i.e., the bacteria and photosensitizers) is assumed to be recovered before the acetic acid purification section. In the separation, the reaction medium is sent to an electrodialysis cell where the acetic acid is concentrated to 70 wt % (ED1).^25^ The concentrated acetic acid solution is then sent to a conventional distillation column (C1), where it reaches the required 99.9 wt % purity. When cysteine is used, the cystine byproduct is separated by filtration before the electrodialysis cell and sent to an electrolyzer (E1), where it is reduced back to cysteine and recycled back to the process.

### Environmental Assessment

The LCA is developed based on the four phases described in the ISO 14040/44 framework.^26^ The first phase consists of defining the goal and scope of the study. In this case, we consider a cradle-to-gate assessment with a cutoff attributional approach of acetic acid synthesis, including all upstream activities, that were retrieved from the Ecoinvent v3.8 database.^27^ The chosen functional unit is 1 kg of acetic acid. The second phase consists of gathering the data required to build the LCIs, which can be found in the Supporting Information (Section B). Here, we define our foreground system for the CO and synthetic methane synthesis and the GTA and SAP routes from the material and energy stream results from the Aspen HYSYS v12 simulations. The BAU route and green H_2_ and DAC CO_2_ were retrieved from the literature. The background system was modeled from activities of the Ecoinvent v3.8 database, including all activities related to raw material and energy production. In the third phase, we compute the LCIA with Brigthway2 version 2.4.2^21^ using the ReCiPe 2016 v1.13 methodology. Finally, in the fourth phase, we interpret the results, which are presented in the Results and Discussion section. In addition, we perform a sensitivity analysis based on 500 Monte Carlo simulations of the backgrounds for all ten scenarios using the Ecoinvent v3.8 pedigree matrix. The approach followed for accounting for carbon removal (i.e., DAC CO_2_ and biogas/biomethane) was to consider a negative contribution when capturing the said carbon from the air and a positive contribution for emitting it back.

### Economic Assessment

We carried out the economic assessment following standard methodologies.^23^ We calculated the total annualized cost per kilogram of acetic acid from the operating (OPEX) and capital (CAPEX) expenditures. More details on the procedure and economic factors employed in the calculations can be found in the Supporting Information (Section C).

## Results and Discussion

The simulation results, both for CO and for acetic acid synthesis, are reported per kg of product in the Supporting Information (Section B). Here, we discuss the environmental and economic results.

### Environmental Results

**Figure 3:**
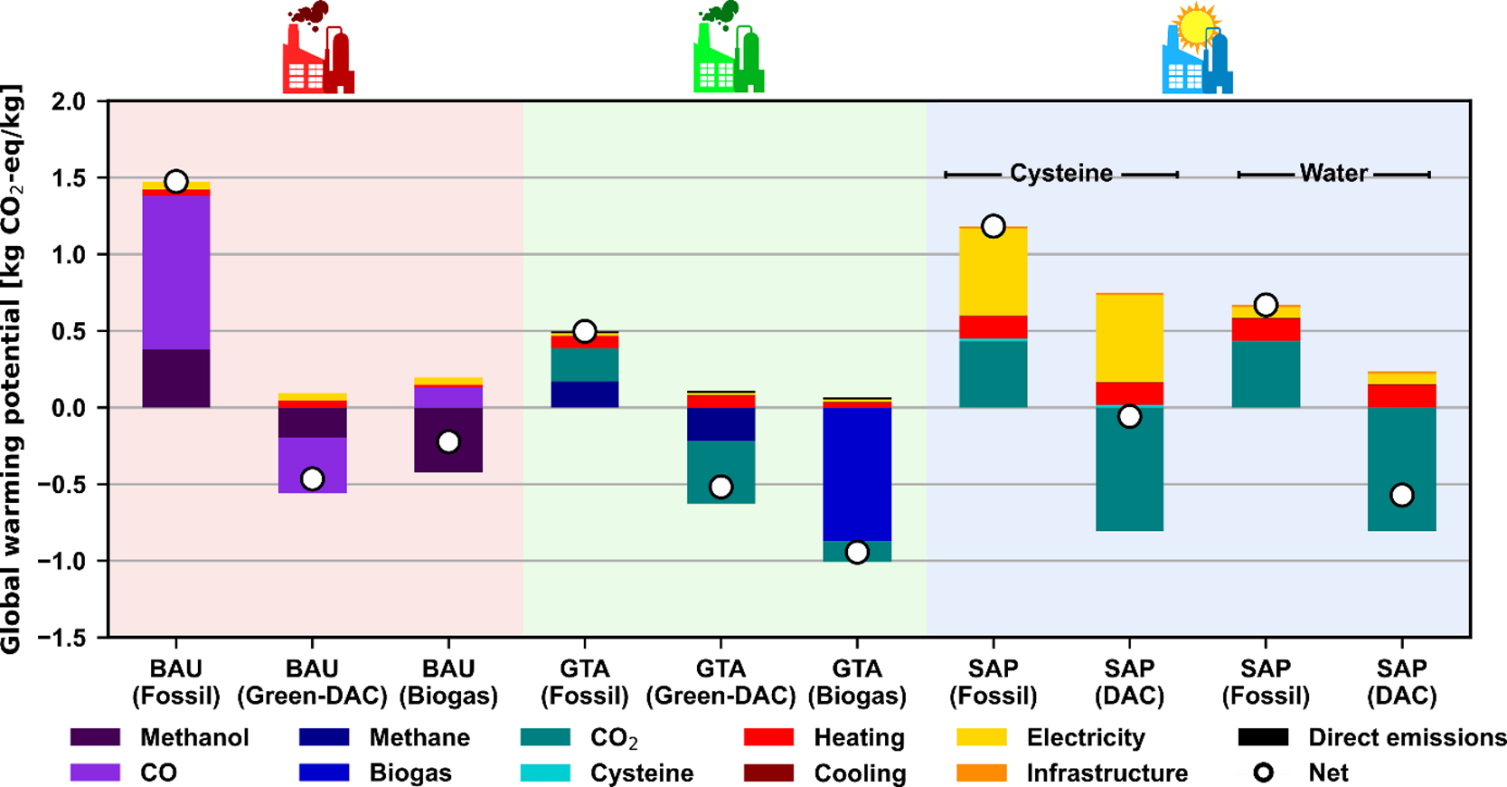
Global warming potential impact results in the synthesis of acetic acid. The “fossil” scenarios assume natural gas and/or coal power plant-captured CO_2_ raw materials; the “green-DAC” and “DAC” scenarios use wind electrolytic H_2_ and/or DAC CO_2_; and the “biogas” scenarios use biomethane or biogas as needed. The “net” contribution represents the overall value of the environmental impact.

In terms of carbon footprint (Figure 3), all evaluated acetic acid synthesis routes outperform the fossil BAU (1.48 kgCO_2_-eq/kg). More specifically, the biogas GTA (−0.92 kgCO_2_-eq/kg) emerges as the best alternative, with an absolute difference of 2.40 kgCO_2_-eq/kg compared to the fossil BAU. The next best option is the DAC SAP using water (−0.57 kgCO_2_-eq/kg), saving 2.05 kgCO_2_-eq/kg in the hypothetical scenario in which it would substitute the BAU. Other interesting routes are the green-DAC BAU (−0.47 kgCO_2_-eq/kg) and GTA (−0.52 kgCO_2_-eq/kg). Both of these routes use electrolytic H_2_ and DAC CO_2_ to synthesize the precursors required for acetic acid production, which are green methanol and green CO for the BAU and green synthetic methane for the GTA. Especially in the case of the BAU variant, this low carbon footprint can prove key in a transition to a more sustainable acetic acid production as it implies conserving the current infrastructure for the synthesis, while the potential better alternatives, such as GTA or SAP, are still at a low technology readiness level (TRL). The remaining technologies with negative cradle-to-gate global warming impact are the biogas BAU (−0.19 kgCO_2_-eq/kg) and the DAC SAP (−0.06 kgCO_2_-eq/kg) using cysteine. The rest of the scenarios, namely, the fossil versions of GTA (0.50 kgCO_2_-eq/kg) and both SAP options (1.18 kgCO_2_-eq/kg with cysteine and 0.67 kgCO_2_-eq/kg with water), show a positive carbon footprint due to the fossil nature of the CO_2_ employed in the synthesis (i.e., captured from a coal carbon plant). However, despite this fact, their carbon footprints still outperform the BAU, showing reductions of 66, 20, and 55%, respectively.

The main contribution that makes fossil BAU the least environmentally friendly alternative is CO (68%), followed by methanol (26%), electricity (3%), and heating (3%). The top 94% of the carbon emissions is attributed to the usage of fossil methane as a raw material in the synthesis of both CO (from the POX of methane followed by cryogenic distillation) and methanol (from the steam reforming of methane-based syngas). In contrast, the green-DAC BAU sees a sharp decrease in the contributions of its main raw materials due to the use of DAC CO_2_ as the carbon source for both the CO (via RWGS with wind electrolytic H_2_) and methanol (via CO_2_ hydrogenation). The biogas BAU, however, shows a different behavior, as CO synthesis by POX of biomethane releases the CO_2_ contained in the biogas, combined with the high impact of air separation O_2_, thus netting a net positive biogenic CO contribution.

The fossil GTA's main contribution is CO_2_ (44%), followed by methane (34%), heating (15%), electricity (3%), direct emissions (3%), and finally cooling (1%). The main contributor here is CO_2_ usage because it is captured from a coal power plant. Hence, no negative contribution is attributed to it, as capturing fossil carbon does not imply any net removal of CO_2_ from the air. Regardless, the net impact of fossil GTA is still three times lower than the fossil BAU. This completely changes when assessing the green-DAC variant of the GTA, where DAC CO_2_ is directly used as a raw material combined with synthetic methane (via the Sabatier reaction of CO_2_ with H_2_), resulting in a net negative contribution comparable to the BAU analogue with green feedstock. However, the best result by far, including all other assessed scenarios, is the GTA route using biogas since its required raw materials perfectly synergize with the composition of biogas (i.e., methane and CO_2_) while even needing additional DAC CO_2_ to carry out the synthesis at an equimolar ratio.

Finally, the fossil CO_2_ SAP routes main contributions are CO_2_ (37 and 65%), electricity (48 and 10%), heating (12 and 22%), infrastructure (1 and 2%), and cysteine (1 and 0%), for the cysteine and water scenarios, respectively. The main difference between both scenarios is attributed to the H_2_ donor and its subsequent treatment. When using cysteine to supply H_2_ for the hydrogenation of CO_2_ to acetic acid, the protein dimerizes into cystine. This cystine then needs to be regenerated back to cysteine, mainly due to the high cost of cysteine of about 600 $/kg. For this task, an electrolytic process is required, leading to the high electricity consumption of the cysteine variant. However, in the scenario of water as the hydrogen donor, this electrolytic process is avoided, resulting in a 43% decrease in the overall carbon footprint of the alternative. The DAC SAP scenarios greatly improve upon these environmental results, making the water variant the second-best evaluated option of all scenarios, only behind the biogas GTA.

**Figure 4:**
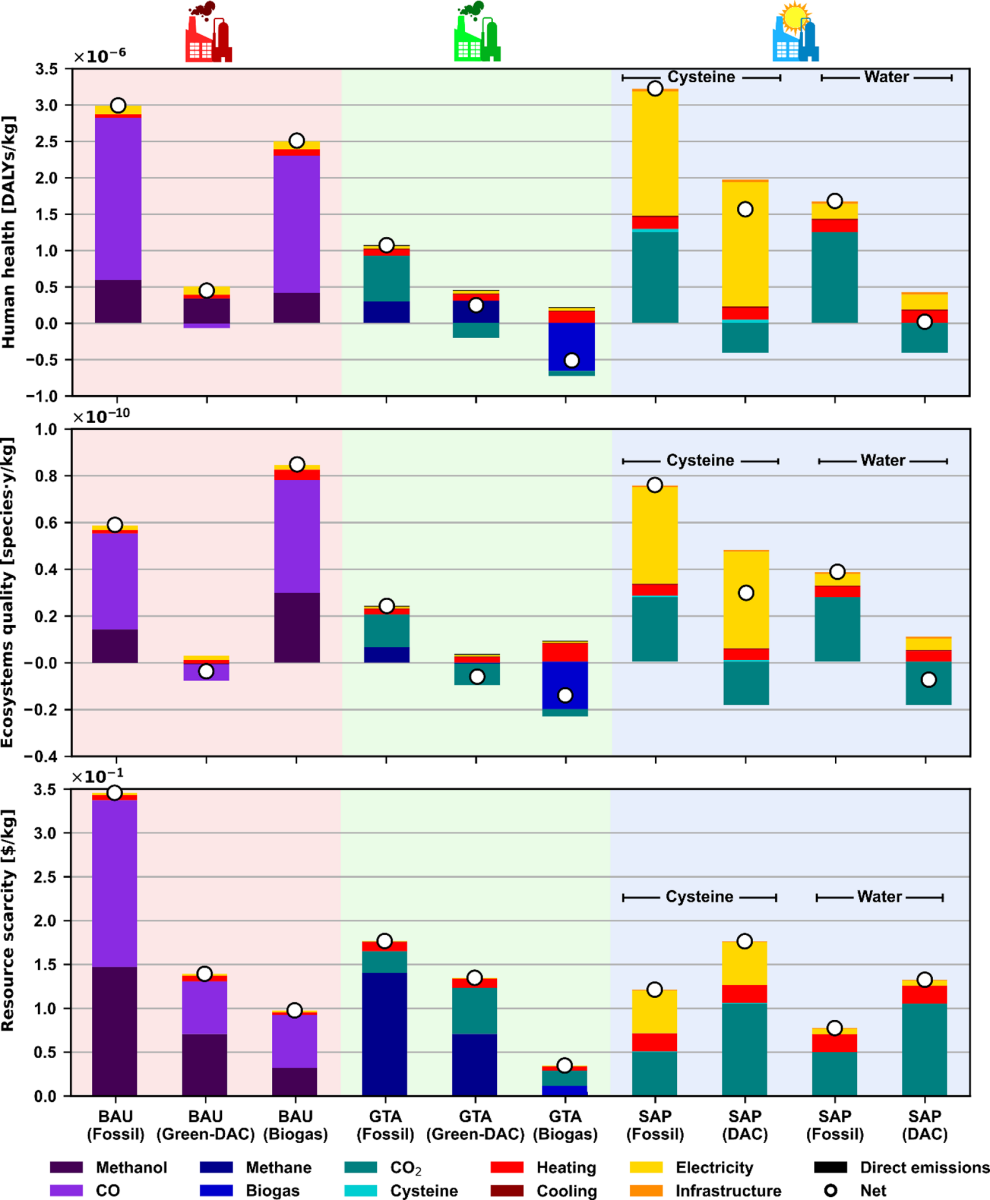
Human health, ecosystem quality, and resource scarcity impact results in the synthesis of acetic acid. The “fossil” scenarios assume natural gas and/or coal power plant-captured CO_2_ raw materials; the “green-DAC” and “DAC” scenarios use wind electrolytic H_2_ and/or DAC CO_2_; and the “biogas” scenarios use biomethane or biogas as needed. The “net” contribution represents the overall value of the environmental impact.

Expanding the analysis to other environmental impacts (Figure 4) shows that all of the assessed scenarios, except for the biogas BAU and the fossil SAP with cysteine, still outperform the fossil BAU when evaluating the damage assessment metrics human health, ecosystem quality, and resource scarcity. Overall, the same trends observed for the global warming impact apply to these metrics, with the biogas GTA being the best alternative, followed by the DAC SAP with water scenario.

As mentioned, the only occurrence of burden shifting is found in the ecosystem quality impact of the biogas BAU scenario and the fossil SAP with cysteine. The first result is a consequence of the biogenic CO_2_ contained in the biogas being emitted after the biomethane separation, while the second stems from the land use required for the solar electricity consumed in the electrolytic cysteine regeneration after the photosynthesis reactor.

The best alternative, the biogas GTA methane carboxylation, reduces the human health, ecosystem quality, and resource scarcity impacts relative to the fossil BAU by 117, 123, and 92%, respectively. In addition, its better performance relative to the green-DAC BAU is also appreciable, with 187, 311, and 79% improvements for the same impact categories. Similar to the GWP results, the steep decrease is a consequence of drastically reducing fossil inputs (mainly natural gas) and substituting them with biogenic CO_2_ and biomethane as the carbon source for the main product synthesis. Furthermore, the energy demand is significantly lowered, since for the BAU route, four reaction steps (i.e., methane reforming to CO, methane reforming to syngas, syngas conversion to methanol, and finally methanol carbonylation to acetic acid) and subsequent separations are required to synthesize acetic acid, while only one step (i.e., direct methane carboxylation to acetic acid) with simple condensation is needed with the GTA technology using biogas.

The second-best alternative, the DAC SAP using water scenario, also sees appreciable reductions of 44, 78, and 35% in human health, ecosystem quality, and resource scarcity, respectively, compared to the fossil BAU. The main contributions to these impacts are energy (i.e., heating and electricity required for the separation of the diluted acetic acid) and the infrastructure (i.e., the materials required for the reactor, such as quartz and aluminum), while DAC CO_2_ pushes the net impacts in human health and ecosystems to negative values due to CO_2_ removal from the atmosphere. On the other hand, the impact related to resources is comparable to the fossil CO_2_ SAP variant due to the energy-intensive, and therefore resource-intensive, DAC process.

**Figure 5:**
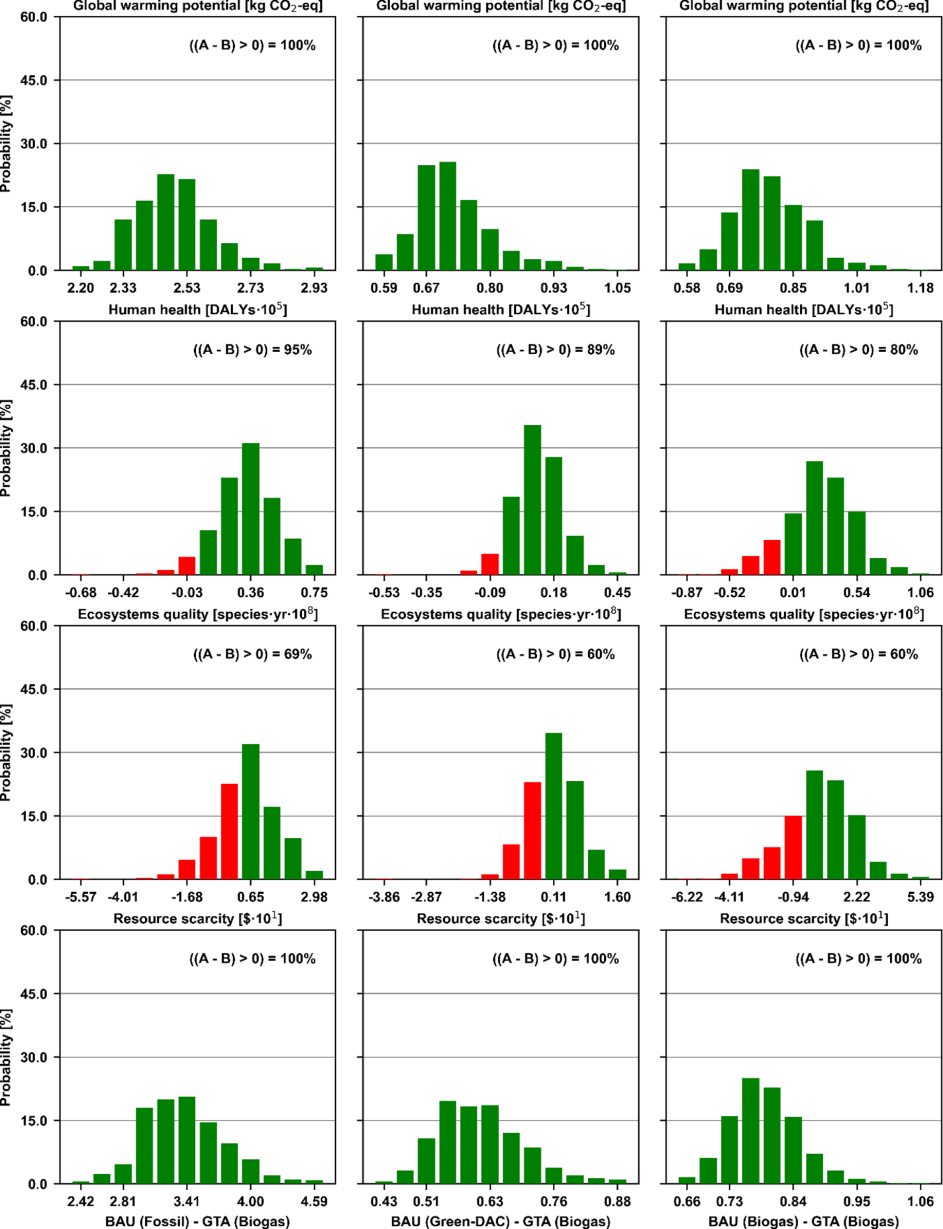
Uncertainty analysis of the three BAU (fossil, green-DAC, and biogas) scenarios (A) minus the GTA (biogas) scenario (B). A result lower than zero is indicative of the occurrence of burden shifting.

To evaluate the effect of uncertainties in the background data on our results, we performed a Monte Carlo analysis for the most promising technology, the GTA using biogas, comparing it against the fossil, green-DAC, and biogas-based BAU processes (Figure 5). For the remaining technologies, this analysis can also be found in the Supporting Information (Section B). Overall, it can be seen that for the climate change, human health, and resource scarcity impacts there is not a statistically relevant probability of burden shifting, while for ecosystem quality, the analysis is not fully conclusive. More specifically, for both climate change and resource scarcity, the GTA using biogas completely outperforms all three BAU variants, with zero probability of burden shifting. Regarding human health, not significant probabilities of 5, 11, and 20% compared to the fossil, green-DAC, and biogas-based BAU are found, respectively. Finally, ecosystem quality results in inconclusive probabilities of burden shifting of 31, 40, and 40% for the same scenarios.

### Economic Assessment

**Figure 6:**
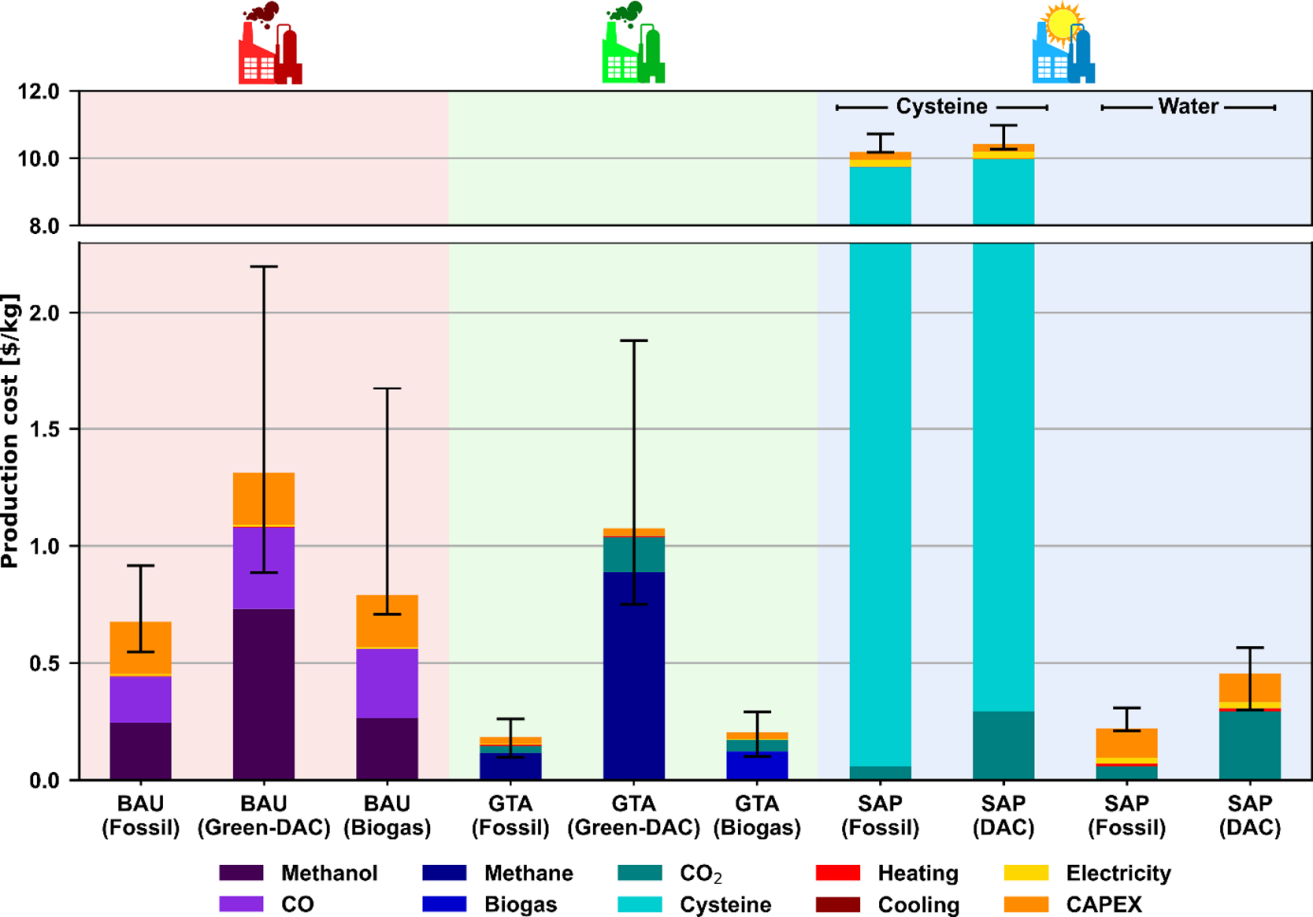
Economic results of the synthesis of acetic acid. The “fossil” scenarios assume natural gas and/or coal power plant-captured CO_2_ raw materials; the “green-DAC” and “DAC” scenarios use wind electrolytic H_2_ and/or DAC CO_2_; and the “biogas” scenarios use biomethane or biogas as needed. Uncertainty in the fossil BAU was adapted from the end of June 2024 global regional reported acetic acid prices. Uncertainty in the rest of the scenarios was considered by varying the prices of the main raw materials (Supporting Information Section D).

As shown in Figure 6, only four synthesis routes outperform the fossil BAU economically (0.68 $/kg): the fossil GTA (0.18 $/kg), the biogas GTA (0.20 $/kg), the fossil SAP, and the DAC SAP both using water (0.30 and 0.53 $/kg, respectively). The main contributor to these costs is the OPEX (67–97%) for all cases, except for the SAP water variants, in which CAPEX comprises 23–48% of the total cost. In contrast, for the GTA scenarios, the CAPEX is very small (0.03 $/kg), only accounting for 3% of the total cost, but in the BAU scenarios, the CAPEX ascends to 17–33% of the total contribution (0.22 $/kg). As depicted in Figure 2b, the GTA process showcases a simple design stemming from easy separation and mild reaction conditions, while the SAP systems (Figure 2c), despite also being relatively simple in design, still need an electrodialysis unit and a distillation column for the more complex separation of the very diluted product. Furthermore, the reactor required for the photosynthesis reaction also appreciably increases the costs, as it is made mainly from expensive borosilicate glass tubes and aluminum rather than standard stainless steel.

As mentioned in the previous section, these routes also present a better environmental performance than the fossil BAU. We note here that we used experimental data generated at the laboratory scale, so the performance will vary well with higher TRLs as these technologies are expected to be deployed at a much larger scale. Specifically, further optimization of these systems can lead to even better results, especially in the case of SAP, where increased quantum efficiencies and conversion values toward acetic acid, currently considered as 1.6 and 1.0%, respectively, would translate into drastically lowering the required reactor size and a less costly separation, rapidly cutting costs even further.

The rest of the scenarios behave as expected, with the green acetic acid scenarios being more expensive compared with their respective fossil alternatives (twice as much for the BAU and 6-fold for the GTA) as it is common in similar systems that use electrolytic H_2_ as feedstock such as green methanol^2^ or green ammonia.^3^ However, special attention should be paid to the SAP systems using cysteine, whose production cost sharply increases to 15 times the cost of the fossil BAU due to the use of the protein as the raw material. This dramatic increase is due to the high price of cysteine (ca. $600/kg), making, despite the high recovery of 99.9% due to the electrolytic regeneration, the system still highly economically unappealing.

## Conclusions

In this work, we assessed ten scenarios for the synthesis of acetic acid, including BAU methanol carbonylation, the novel GTA methane carboxylation, and SAP using cysteine and water as the hydrogen donors for the system. Furthermore, we studied the performance of these synthesis routes under different fossil and renewable feedstocks.

We found two potential economic and environmental win–win scenarios: the biogas-based GTA process and the SAP route using DAC CO_2_ and water as a hydrogen donor. These alternatives present, respectively, 2.40 and 1.93 kgCO_2_-eq/kg less carbon emissions and a 70 to 14% reduction in production cost compared to the fossil BAU (1.48 kgCO_2_-eq/kg and 0.68 $/kg). Furthermore, when extending the analysis to other LCA metrics for the best-performing technology, the GTA using biogas, no signs of burden shifting are detected in resource scarcity, while the probability of burden shifting is not significant in human health and inconclusive in ecosystem quality. These drastic improvements are due to simpler reaction systems that can produce acetic acid in a single step rather than the original four steps required in the BAU methanol carbonylation, which leads to significant energy and material savings.

Our results highlight the potential benefits of exploring nonconventional synthesis routes for chemicals. More specifically, synthesis routes with higher atom efficiency in fewer reaction steps could play a key role in the transition toward a more sustainable chemical sector by lowering both the energy and material demand to ultimately reduce costs and impacts.

## Supporting Information Available

Design of the simulations (Section A), additional environmental results (Section B), economic analysis parameters (Section C), and assumptions (Section D) (PDF)

The data that support the findings of this study are openly available in Zenodo at https://doi.org/10.5281/zenodo.14671903.

## Abbreviations

BAU: business-as-usual
CyS: cysteine
CySS: cystine
DAC: direct air capture
DMR: dry methane reforming
GTA: gas-to-acid
POX: partial oxidation
LCA: life-cycle assessment
LCI: life-cycle inventory
LCIA: life-cycle impact assessment
PVA: poly(vinyl alcohol)
RWGS: reverse water–gas shift
SAP: semiartificial photosynthesis
SMR: steam methane reforming
TRL: technology readiness level
VCM: vinyl chloride monomer
