# Supplementary material for: Win–Win More Sustainable Routes for Acetic Acid Synthesis
Source: ACS Sustain Chem Eng. 2025 Jan 22;13(4):1522–31. doi: 10.1021/acssuschemeng.4c07324 (PMC11795521; doi:10.1021/acssuschemeng.4c07324)
Supplement: Supplementary file 1 — sc4c07324_si_001.pdf [file sc4c07324_si_001.pdf]

Supporting information

Win-Win more Sustainable Routes for Acetic Acid Synthesis

Juan D. Medrano-García[*,†], *Raúl Calvo-Serrano*[††], *Haining Tian*[†††] *and Gonzalo Guillén-Gosálbez*[*,†]

[†] Institute for Chemical and Bioengineering, Department of Chemistry and Applied Biosciences, ETH Zurich, Vladimir Prelog Weg 1, 8093 Zurich, Switzerland.

[††] Departament d'Enginyeria Química i Ciència de Materials, Institut Quimic de Sarrià, Universitat Ramon Llull, Via Augusta 390, 08017 Barcelona, Spain

[†††] Department of Chemistry─Ångström laboratory, Physical Chemistry, Uppsala University, Box 521, 75120, Uppsala, Sweden

* Corresponding author.

E-mail address: juan.diego.medrano@chem.ethz.ch

E-mail address: gonzalo.guillen.gosalbez@chem.ethz.ch.

This document includes additional material to the content presented in the main article. Here we describe the process simulations, the computation of the life cycle inventories, further environmental results, and the data used in the economic analysis.

Number of pages: 51

Number of figures: 11

Number of tables: 27

A. Process simulations

We developed a range of simulations to support the economic and environmental assessment calculations. We use the NRTL and Peng Robinson thermodynamic packages in Aspen Plus v12 to model the systems as suggested by the literature[1]. Absorber columns were converged using the amines package. Unless stated otherwise, streams are fed to distillation columns at their dew point (vapor streams) or bubble point (liquid streams) and reactors are operated isothermally. Compressors assume 0.72 isoenthropic efficiencies and compression ratios (r) are calculated as a function of the inlet pressure (P_{in}), outlet pressure (P_{out}) and number of stages (n) (Eq.(S1)). Finally, we compute the minimum utility consumption with Aspen Energy Analyzer. The inventories resulting from the material and energy flows of the simulations are presented in **Section B**.

$$r = \left(\frac{P_{out}}{P_{in}} \right)^n \qquad \text{(S1)}$$

A.1. Carbon monoxide production from methane partial oxidation (POX)

Carbon monoxide is one of the main raw materials in the business-as-usual (BAU) methanol carboxylation acetic acid synthesis. Hence, for the fossil and bio BAU scenarios, we developed a BAU CO simulation based on partial oxidation (POX) of methane with subsequent cryogenic distillation (**Figure S1**)[2]. The process starts with methane and O_2 at 25 ºC and 1 bar and 2:1 molar ratio being compressed to 30 bar in three stages with intercooling at 40 ºC and heated up to 800 ºC before entering the adiabatic POX reactor (R-100), modeled as a Gibbs equilibrium reactor. The resulting syngas is cooled down to 40 ºC to condense most of the water (FS-100) and then sent to an amine absorber with 10 stages (C-100) to remove the CO_2. The absorbent liquid employed is 30 wt.% methyl ethyl amine (MEA) at 25 ºC and the absorption is carried out at 30 bar. The CO_2 is removed from the solution in a 30-stage stripper column (C-101) operating at 2 bar and with the reboiler at 124 ºC and the CO_2 leaving the top of the column at 90 ºC. After a 0.1 % purge, the amine solution is recompressed to 30 bar and recycled back to the absorption tower, while the CO_2 is vented. The syngas stream recovered from the absorber is assumed to be sent to a molecular sieve (MS-100), modeled as a component splitter, to remove the remaining traces of water. After drying the syngas, it is cooled down to -190 ºC and sent to a flash separator (FS-101) where a 53 wt.% H_2 stream with unseparated CO is recovered as vapor and then burnt with stoichiometric air in a furnace to produce energy for the process. The liquid fraction, containing 95 wt.% CO and the unreacted methane, is heated up to saturation temperature (-142 ºC) and sent to a 24-stage cryogenic distillation column (feed at 9th stage, distillate recoveries of 99.9% CO and 10% methane, reflux ratio 0f 1.06 and energy consumption of 1.12 MW at the reboiler and 0.92 MW at the condenser) (C-102), where CO at 99 wt.% is recovered at the top at -146 ºC and a 98 wt.% methane-rich stream (-97 ºC) is heated up to 25 ºC and mixed with the compressed fresh methane feed of the POX reactor. The heat

integration analysis was divided between cryogenic streams (cold box) and the rest of the process.

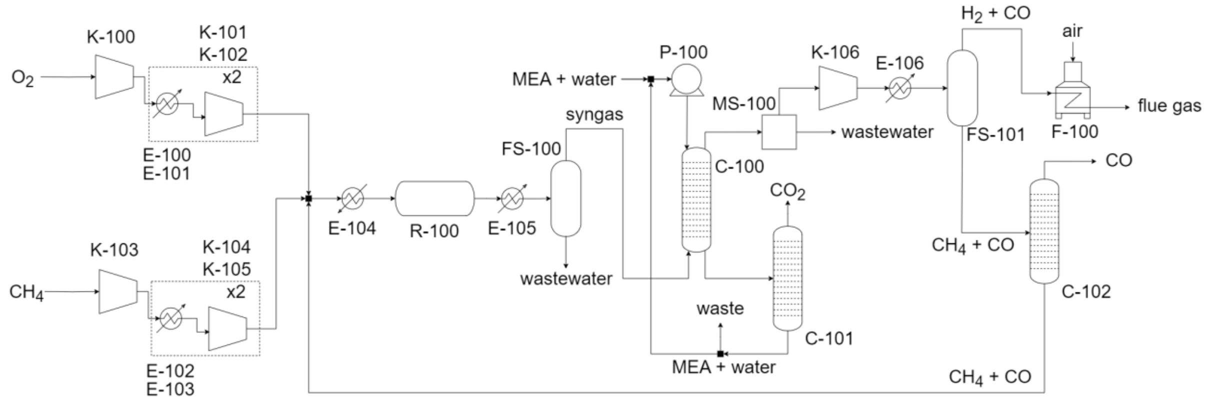

Figure S1. Carbon monoxide synthesis by POX of methane simplified flowsheet.

A.2. Green carbon monoxide production from captured CO_2 and electrolytic H_2 using reverse water gas shift (RWGS)

Green carbon monoxide for the DAC-green BAU scenario was assumed to be produced directly from CO_2 and H_2 *via* the RWGS reaction (**Figure S2**)[2]. This process assumes the use of proton exchange membrane (PEM) electrolytic H_2 that comes out of the electrolyzer at 80 ºC and 30 bar and direct air capture (DAC) CO_2 at 1 bar and 25 ºC. The feedstock is fed to the reactor at a 1:1 molar ratio. The CO_2 stream is compressed from 1 bar to 9 bar in two stages with intercooling to 40 ºC (compression ratio of 3.00), while the H_2 stream is the decompressed 9 bar. The reaction is carried out at 9 bar and 800 ºC in an isothermal Gibbs equilibrium reactor (R-100). The product syngas is cooled down to 40 ºC and sent to a flash separator (FS-100) to remove the water as the liquid stream. The dried syngas (0.33:0.33:0.33 molar ratio of H_2, CO and CO_2) then is sent to a pressure swing adsorption (PSA) unit (PSA-100), modeled as a component splitter, where 90% of the CO is recovered as the product at 99 wt.% purity and 99% of the reject stream is recycled back to the RWGS reactor. The remaining 1% is sent to a furnace and combusted with stoichiometric air.

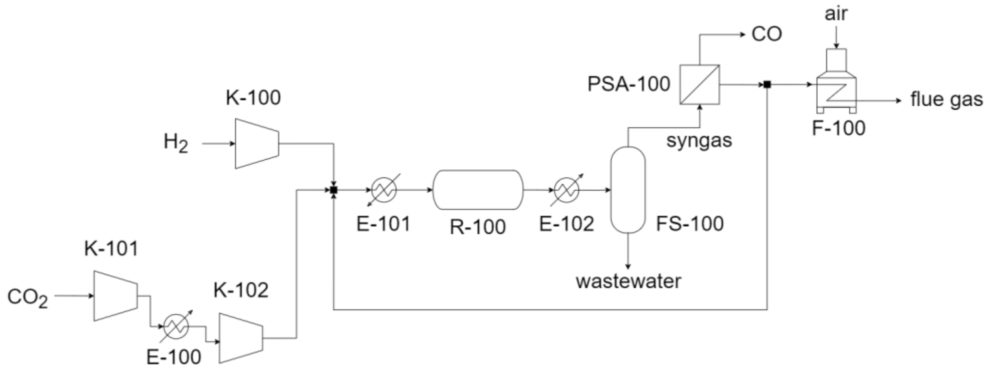

Figure S2. Carbon monoxide synthesis by RWGS of DAC CO_2 and green H_2 simplified flowsheet.

A.3. Synthetic methane production from captured CO_2 and electrolytic H_2 using the Sabatier reaction

The production of synthetic methane for the DAC-green gas-to-acid (GTA) scenario was modeled using the Sabatier reaction with DAC CO_2 (1 bar, 25 ºC) and electrolytic H_2 (30 bar, 80 ºC) as raw materials (**Figure S3**). First, CO_2 is compressed in two stages with intercooling at 40 ºC (compression ratio of 5.48). Then, the two gas streams are mixed at a 4:1 molar ratio of H_2 and CO_2 and heated up to the reaction temperature (300 ºC). The Sabatier reactor was modeled as an adiabatic equilibrium Gibbs reactor (R-100). The product stream is then cooled down to 40 ºC and, after a small purge (0.1%), sent to a flash separator (FS-100) where most of the water is condensed and removed. Then, the stream containing the produced methane, byproduct CO and unreacted syngas is sent to an ideal component splitter unit (I-100) where all methane is recovered and the unreacted CO_2 and H_2 with the by-product CO are recycled back to the reactor. The purge is combusted stoichiometrically with air (F-100) to supply energy for the process.

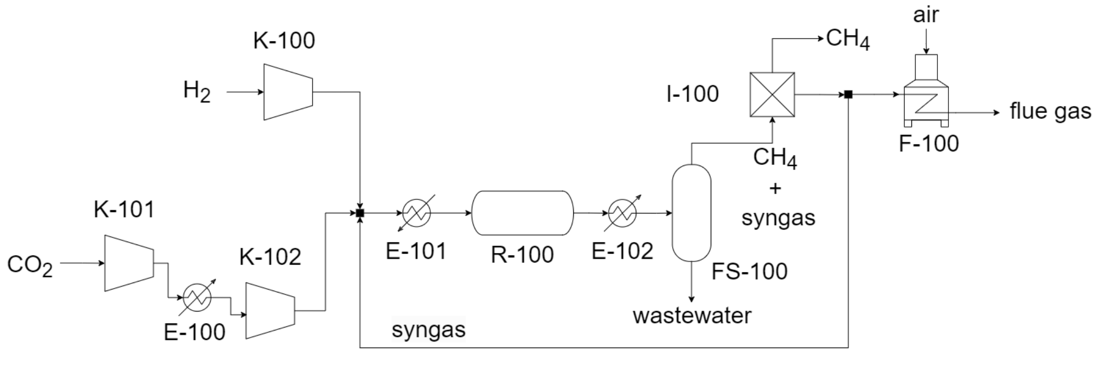

Figure S3. Methane production from DAC CO_2 and green H_2 simplified flowsheet.

A.4. Gas-to-acid (GTA) acetic acid production from methane and CO_2

The methane carboxylation process uses a 1:1 molar feed ratio of methane and CO_2 (**Figure S4**). The feedstock (1 bar, 25 ºC) is compressed to 2 bar in one stage (compression ratio of 1.00), heated up to 300 ºC and sent to the reactor, modeled as an isothermal conversion reactor. The conversion was set to 8.3% of methane to acetic acid following Eq.(S2):

$$CH_4 + CO_2 \rightarrow CH_3COOH \quad \text{(S2)}$$

After the reaction, the product stream is cooled down to 5 ºC and sent to a flash separator, here, acetic acid is removed at 99.99 wt.% purity and the unreacted raw materials are recycled back to the inlet of the reactor after a 0.1% purge. The purged gases are combusted with stoichiometric air to supply energy to the process.

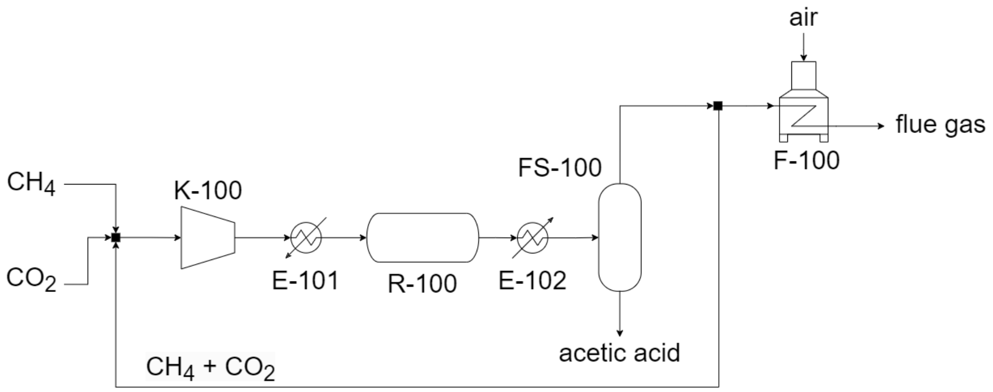

Figure S4. Acetic acid synthesis using methane and CO_2 simplified flowsheet.

A.5. Semi-artificial photosynthesis (SAP) acetic acid production from cysteine/water and CO_2

In this process, CO_2, cysteine ($C_3H_7NO_2S$) and the reaction medium, assumed to be water (molar ratio 60:6:1000), are sent (1 bar, 25 ºC) to the photosynthesis reactor, modeled as an isothermal conversion reactor (**Figure S5**). The conversion was set to 1% of CO_2 to acetic acid following Eq.(S3)[3]:

$$2CO_2 + 8C_3H_7NO_2S \rightarrow CH_3COOH + 4C_6H_{12}N_2O_4S_2 + 2H_2O \quad \text{(S3)}$$

The temperature in the reactor (60 ºC) is considered to be reached just by sunlight irradiation. Then, the mixture is sent to a flash separator where the CO_2 is removed and recycled back to the inlet of the reactor. Cystine ($C_6H_{12}N_2O_4S_2$) by-product is assumed to precipitate and is sent to the electrolyzer where it is regenerated back to cysteine with a 50% energy efficiency. Cysteine losses were assumed to be 0.1% of the reactor feed. The liquid phase, which contains the diluted acetic acid at 100 ppm concentration, is sent to the electrodialysis unit where it is concentrated to 70 wt.%[4] assuming an energy consumption of 0.61 kWh/kg[5]. The reject stream (medium) is sent back to the reactor inlet, while the concentrated acetic acid solution is finally sent to a 43-stage distillation column working at atmospheric pressure (stage 25 feed, 2.4 reflux ratio, 99.7 % recovery of water in distillate and 0.1% recovery of acetic acid in distillate). Acetic acid is recovered as the bottom product at 99.9 wt.% purity and 117 ºC and water (the remaining medium) as the top product at 100 ºC, which is cooled down and sent back to the reactor.

The water variant of this process, i.e., no cysteine is required, considers a similar configuration but omits the cysteine regeneration section and assumes that the source of H_2 for acetic acid synthesis comes from water (Eq.(S4)) with also a 1% CO_2 conversion:

$$2CO_2 + 2H_2O \rightarrow CH_3COOH + 2O_2 \quad \text{(S4)}$$

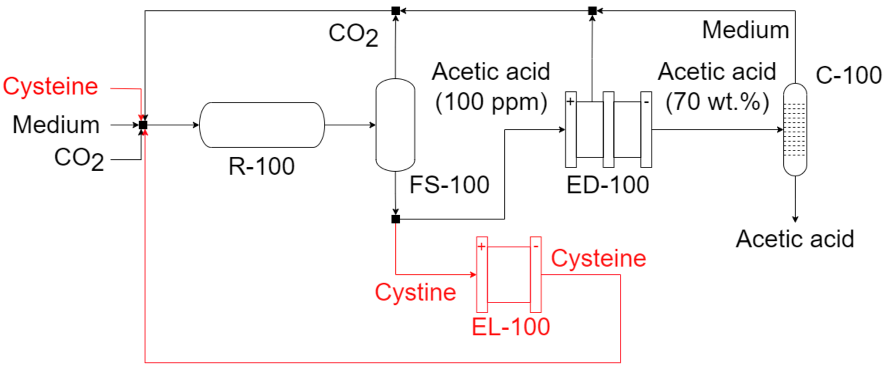

Figure S5. Acetic acid synthesis production by semi-artificial photosynthesis simplified flowsheet. Red lines indicate the modifications implemented to use cysteine as electron donor.

The reactor was sized using Eq.(S5):

$$A = \frac{\Delta H_r \cdot N}{I \cdot \eta} \cdot 1000 \quad (5)$$

Where A is the surface area of the reactor borosilicate tubes in m^2, ΔH_r is the reaction enthalpy (168943.5 kJ/kmol) of reaction (2) at 60 ºC (estimated with Aspen Plus v12), N is the molar flow of acetic acid (0.12 kmol/s) assuming an industrial capacity of 25 t/h, η is the quantum efficiency or fraction of the solar energy used in the photosynthesis process (0.016)[3], I is the sun mean daily annual irradiance of Seville assuming the month of December is used for maintenance (228.94 W/m^2)[6]. The mass of borosilicate was calculated assuming an internal diameter of 4.0 cm and external diameter of 10.5 cm per tube and a density of 2200 kg/m^3.

The electricity required in the cysteine regeneration was estimated considering an overall 50% energetic efficiency and assuming that the standard Gibbs free energy of the overall reaction represents the energy consumption in the ideal regeneration scenario (Eq.(S6)):

$$\Delta G^0 = -E_{cell} \cdot n \cdot F \quad \text{(S6)}$$

Where ΔG^0 (J/(2 moles cysteine)) is the standard Gibbs free energy of the overall regeneration reaction (Eq.(S7)), E_{cell} is the potential of the cell (V), calculated as the potential of the cathode (-0.22 V vs standard hydrogen electrode, SHE)[7] (Eq.(S8)) minus the potential of the anode (0.404 V vs SHE at pH 14) (Eq.(S9)), n is the transferred number of electrons in the reaction and F is the faradaic constant (96485 s·A/mol).

$$C_6H_{12}N_2O_4S_2 + H_2O \rightarrow 2C_3H_7NO_2S + 0.5O_2 \quad \text{(S7)}$$

$$C_6H_{12}N_2O_4S_2 + 2H^+ + 2e^- \rightarrow 2C_3H_7NO_2S \quad \text{(S8)}$$

$$H_2O \rightarrow 0.5O_2 + 2H^+ + 2e^- \quad \text{(S9)}$$

From an intermittency point of view, both the cysteine regeneration unit (when using cysteine as electron donor) and the electrodialysis unit would require energy storage to continue to operate during the hours when sunlight is unavailable, since the photosynthesis reactor will continue to produce acetic acid even during the dark cycle[8]. Based on a solar capacity factor of 21% (mean value of Seville, Spain during the months of January to November)[16], we consider the installation of Li-ion NMC111 batteries to include the energy storage required by the SAP systems. These batteries are assumed to possess a gravimetric energy density of 0.18 kWh/kg and a lifetime of 1500 cycles[15]. Hence, the mass of battery required to operate the SAP plant is calculated as the total electricity required by the cysteine regeneration electrolyzer and the electrodialysis unit multiplied by the fraction of time when solar electricity is unavailable (0.79) by the inverse of the battery gravimetric energy density (5.56 kg/kWh).

A.6. Process stream data

Table S1. Major process stream data for the carbon monoxide synthesis by POX of methane (fossil BAU scenario) simulation (**Figure S1**).

Stream	R-100 (in)	R-100 (out)	FS-101 (in)	FS-101 (bottom)	C-102 (in)	C-102 (top)
**Temperature [°C]**	800	1194	-190	-190	-142	-146
**Pressure [bar]**	30	30	30	30	30	30
**Mass flow [kg/h]**	$1.63 \cdot 10^4$	$1.63 \cdot 10^4$	$1.55 \cdot 10^4$	$1.22 \cdot 10^4$	$1.22 \cdot 10^4$	$1.17 \cdot 10^4$
**Mass fraction**						
**H_2**	-	0.11	0.12	-	0.00	0.00
**CO_2**	-	0.02	-	-	-	
**CO**	-	0.80	0.84	0.95	0.95	0.99
**H_2O**	-	0.03	-	-	-	
**CH_4**	0.50	0.03	0.04	0.05	0.05	0.00
**O_2**	0.50	-	-	-	-	

Table S2. Major process stream data for the carbon monoxide synthesis simulation by reverse water gas shift (RWGS) of captured CO_2 and electrolytic H_2 (green BAU scenario) (**Figure S2**).

**Stream**	**R-100 (in)**	**R-100 (out)**	**PSA-100 (in)**	**PSA-100 (product)**
**Temperature [ºC]**	800	800	60	60
**Pressure [bar]**	9	9	9	1
**Mass flow [kg/h]**	$4.19\cdot10^4$	$4.19\cdot10^4$	$3.44\cdot10^4$	$1.17\cdot10^4$
**Mass fraction**				
**H_2**	0.04	0.02	0.03	0.00
**CO_2**	0.92	0.49	0.59	0.00
**CO**	0.03	0.31	0.37	0.99
**H_2O**	0.00	0.18	0.00	-

Table S3. Major process stream data for the synthetic methane production from captured CO_2 and electrolytic H_2 via the Sabatier reaction (green GTA scenario) (**Figure S3**).

**Stream**	**R-100 (in)**	**R-100 (out)**	**FS-100 (top)**
**Temperature [ºC]**	300	300	40
**Pressure [bar]**	30	30	30
**Mass flow [kg/h]**	$5.25 \cdot 10^3$	$5.25 \cdot 10^4$	$1.65 \cdot 10^4$
**Mass fraction**			
**H_2**	0.16	0.00	0.01
**CO_2**	0.84	0.01	0.02
**CO**	0.00	0.00	0.00
**H_2O**	0.00	0.69	0.00
**CH_4**	-	0.30	0.97

Table S4. Major process stream data for the gas-to-acid (GTA) synthetic acetic acid production from captured CO_2 and methane (**Figure S4**).

**Stream**	**R-100 (in)**	**R-100 (out)**	**FS-100 (bottom)**
**Temperature [ºC]**	300	300	5
**Pressure [bar]**	2	2	30
**Mass flow [kg/h]**	$1.59 \cdot 10^5$	$1.59 \cdot 10^5$	$2.50 \cdot 10^4$
**Mass fraction**			
**CO_2**	0.49	0.38	0.01
**CH_4**	0.50	0.46	0.00
**CH_3COOH**	0.01	0.16	0.99

Table S5. Major process stream data for the semi-artificial photosynthesis (SAP) acetic acid production from cysteine and CO_2 (**Figure S5**).

**Stream**	**R-100 (in)**	**R-100 (out)**	**EL-100 (in**	**EL-100 (out)**	**ED-100 (in)**	**C-100 (in)**	**C-100 (bot.)**
**Temperature [°C]**	60	60	60	60	60	105	117
**Pressure [bar]**	1	1	1	1	1	1	1
**Mass flow [kg/h]**	$2.87 \cdot 10^7$	$2.87 \cdot 10^7$	$2.87 \cdot 10^7$	$2.87 \cdot 10^7$	$2.50 \cdot 10^7$	$3.57 \cdot 10^4$	$2.50 \cdot 10^4$
**Mass fraction**							
**CO_2**	0.12	0.12	0.12	0.12	1.00	-	-
**H_2O**	0.84	0.84	0.84	0.84	-	0.30	0.00
**cysteine**	0.03	0.02	0.02	0.02	0.00	-	-
**cystine**	-	0.01	0.01	0.01	-		
**CH_3COOH**	-	0.00	0.00	0.00	0.00	0.70	1.00

Table S6. Major process stream data for the semi-artificial photosynthesis (SAP) acetic acid production from water and CO_2 (**Figure S5**).

**Stream**	**R-100 (in)**	**R-100 (out)**	**ED-100 (in)**	**C-100 (in)**	**C-100 (bottom)**
**Temperature [ºC]**	60	60	60	105	117
**Pressure [bar]**	1	1	1	1	1
**Mass flow [kg/h]**	28688688	28688688	25031232	35746	25029
**Mass fraction**					
**CO_2**	0.13	0.13	1.00	-	-
**H_2O**	0.87	0.87	-	0.30	0.00
**CH_3COOH**	-	0.00	0.00	0.70	1.00
**O_2**	-	0.00	-	-	

B. Life cycle assessment (LCA)

In this section, we present the main results of the simulations as the net material and energy streams per functional unit used, i.e., life cycle inventories (LCIs), for the environmental and economic analyses. Then, we show the Monte Carlo analysis results (ReCiPe 2016 v1.1 endpoints) for all the studied scenarios.

B.1. Life cycle inventories (LCIs)

In this section, we present the main results of the simulations (net material and energy streams per kg of product for chemicals, and MJ of energy for utilities) in the form of LCIs. Furthermore, we also show the inventories for the BAU scenario, cryogenic refrigeration and DAC CO_2 built from literature data. Biogas was considered as an European mix with 34% of the contributions coming from animal manure, 26% from agricultural residues, 23% from sequential crops, 10% from industrial wastewater, 5% from biowaste and 2% from sewage sludge. In general, the activities contribution to each LCI were chosen prioritizing global (GLO) regionalization when available, followed by “rest-of-the-world” (RoW) and Europe (RER). SAP systems were located in Seville, Spain, and, as such, activities were chosen prioritizing Spain (ES), Europe (RER), rest-of-the-world (RoW) and global regionalization (GLO), respectively.

Table S7. Carbon monoxide synthesis by POX of methane (fossil BAU scenario) LCI.

Functional unit: 1 kg CO		
**Input**	**Amount**	**Units**
Natural gas (market group for natural gas, high pressure)	$9.17 \cdot 10^{-1}$	m^3
Oxygen (market for oxygen, liquid)	$6.98 \cdot 10^{-1}$	kg
Water (market group for tap water)	$1.61 \cdot 10^{-2}$	kg
Monoethanolamine (market for monoethanolamine)	$1.21 \cdot 10^{-3}$	kg
Heating (market for heat, district or industrial, natural gas)	$1.64 \cdot 10^{-1}$	MJ
Cooling (water from 20 to 25 ºC)[9]	$3.06 \cdot 10^{0}$	MJ
Refrigeration (–195 ºC) (**Table S10**)	$2.4 \cdot 10^{-1}$	MJ
Electricity (market for electricity, high voltage)	$7.85 \cdot 10^{-2}$	kWh
**Output**		
Water (emission to water)	$2.17 \cdot 10^{-7}$	m^3
Water (emission to water)	$6.01 \cdot 10^{-5}$	m^3
Carbon dioxide (emission to air)	$2.28 \cdot 10^{-2}$	kg
Monoethanolamine (emission to water)	$1.11 \cdot 10^{-3}$	kg
Monoethanolamine (emission to air)	$9.67 \cdot 10^{-5}$	kg
Carbon monoxide (emission to air)	$1.46 \cdot 10^{-6}$	kg
Hydrogen (emission to air)	$9.70 \cdot 10^{-6}$	kg

Table S8. Carbon monoxide synthesis by POX of biomethane (bio BAU scenario) LCI.

**Functional unit: 1 kg CO**		
**Input**	**Amount**	**Units**
Biomethane (market for biomethane, 24 bar w/ CCS)[10]	$9.17 \cdot 10^{-1}$	m^3
Oxygen (market for oxygen, liquid)	$6.98 \cdot 10^{-1}$	kg
Water (market group for tap water)	$1.61 \cdot 10^{-2}$	kg
Monoethanolamine (market for monoethanolamine)	$1.21 \cdot 10^{-3}$	kg
Heating (biomethane) (**Table S11**)	$1.64 \cdot 10^{-1}$	MJ
Cooling (water from 20 to 25 ºC)[9]	$3.06 \cdot 10^{0}$	MJ
Refrigeration (–195 ºC) (**Table S10**)	$2.4 \cdot 10^{-1}$	MJ
Electricity (market for electricity, high voltage)	$7.85 \cdot 10^{-2}$	kWh
**Output**		
Water (emission to air)	$2.17 \cdot 10^{-7}$	m^3
Water (emission to water)	$6.01 \cdot 10^{-5}$	m^3
Carbon dioxide (emission to air)	$2.28 \cdot 10^{-2}$	kg
Monoethanolamine (emission to water)	$1.11 \cdot 10^{-3}$	kg
Monoethanolamine (emission to air)	$9.67 \cdot 10^{-5}$	kg
Carbon monoxide (emission to air)	$1.46 \cdot 10^{-6}$	kg
Hydrogen (emission to air)	$9.70 \cdot 10^{-6}$	kg

Table S9. Green carbon monoxide synthesis by RWGS of green H_2 and DAC CO_2 (green-DAC BAU scenario) LCI.

Functional unit: 1 kg CO		
**Input**	**Amount**	**Units**
Carbon dioxide (direct air capture) (**Table S14**)	1.58	kg
Hydrogen (electrolysis with wind electricity)[11]	$7.27·10^{-1}$	kg
Heating (market for heat, district or industrial, natural gas)	$3.26·10^{-1}$	MJ
Cooling (water from 25 to 20 ºC)[9]	$2.03·10^{0}$	MJ
Electricity (market for electricity, high voltage)	$6.50·10^{-2}$	kWh
**Output**		
Water (emission to air)	$1.62·10^{-6}$	m^3
Water (emission to water)	$6.46·10^{-4}$	m^3
Carbon dioxide (emission to air)	$1.92·10^{-2}$	kg
Hydrogen (emission to air)	$5.70·10^{-8}$	kg

Table S10. Refrigeration cascade cycle propylene-ethylene-methane-N_2 (from -195 ºC to -190 ºC) LCI[12].

Functional unit: 1 MJ cooling		
**Input**	**Amount**	**Units**
Cooling (water from 25 to 20 ºC)[9]	$1.71 \cdot 10^{1}$	MJ
Electricity (market for electricity, high voltage)	$4.70 \cdot 10^{0}$	kWh

Table S11. Heating from biomethane (from 1000 to 900 ºC) LCI.

Functional unit: 1 MJ heating		
**Input**	**Amount**	**Units**
Biomethane (market for biomethane, 24 bar w/ CCS)[10]	$5.13 \cdot 10^{-2}$	m^3
**Output**		
Water (emission to air)	$5.34 \cdot 10^{-10}$	m^3
Water (emission to water)	$5.27 \cdot 10^{-5}$	m^3
Carbon dioxide (emission to air)	$9.57 \cdot 10^{-2}$	kg
Carbon monoxide (emission to air)	$4.73 \cdot 10^{-9}$	kg
Hydrogen (emission to air)	$2.57 \cdot 10^{-5}$	kg

Table S12. Synthetic methane from Sabatier reaction LCI.

Functional unit: 1 kg methane		
**Input**	**Amount**	**Units**
Carbon dioxide (direct air capture) (**Table S14**)	$2.94·10^{0}$	kg
Hydrogen (electrolysis with wind electricity)[11]	$5.10·10^{-1}$	kg
Cooling (water from 25 to 20 ºC)[9]	$1.08·10^{1}$	MJ
Electricity (market for electricity, high voltage)	$3.30·10^{-1}$	kWh
**Output**		
Carbon dioxide (emission to air)	0.19	kg

Table S13. Cysteine production from acid hydrolyzation of poultry feathers LCI.

Functional unit: 1 kg cysteine[13]		
**Input**	**Amount**	**Units**
HCl 30% (market for hydrochloric acid, without water, 30% solution)	$5.25·10^{-1}$	kg
Water (market group for tap water)	$4.50·10^{-2}$	kg
Activated carbon (market for activated carbon, granular)	$4.00·10^{-3}$	kg
NaOH 50% (market for sodium hydroxide, without water, 50% solution)	$3.84·10^{-1}$	kg
NH_3 (market for ammonia, anhydrous, liquid)	$7.78·10^{-3}$	kg

Table S14. Direct air captured (DAC) CO_2 LCI[14].

Functional unit: 1 kg captured CO_2		
**Input**	**Amount**	**Units**
Water (market for tap water)	$3.1 \cdot 10^{0}$	kg
Natural gas (market for natural gas, high pressure)	$1.92 \cdot 10^{-1}$	m^3
Calcium carbonate (market for calcium carbonate, precipitated)	$1.99 \cdot 10^{-2}$	MJ
Electricity (market for electricity, high voltage)	$1.49 \cdot 10^{-1}$	kWh
**Output**		
Carbon dioxide (emission to air)	$-7.43 \cdot 10^{-1}$	kg

Table S15. Acetic acid synthesis by methanol carbonylation (fossil BAU scenario) LCI.

Functional unit: 1 kg acetic acid		
**Input**	**Amount**	**Units**
Methanol (market for methanol)	$5.44 \cdot 10^{-1}$	kg
Carbon monoxide (**Table S7**)	$4.91 \cdot 10^{-1}$	kg
Water (market group for tap water)	$8.20 \cdot 10^{-1}$	kg
Heating (market for heat, district or industrial, natural gas)	$1.05 \cdot 10^{0}$	MJ
Electricity (market for electricity, high voltage)	$7.01 \cdot 10^{-2}$	kWh

Table S16. Green acetic acid synthesis by green methanol carbonylation (green-DAC BAU scenario) LCI.

Functional unit: 1 kg acetic acid		
**Input**	**Amount**	**Units**
Green methanol[15]	$5.44\cdot10^{-1}$	kg
Green carbon monoxide (**Table S9**)	$4.91\cdot10^{-1}$	kg
Water (market group for tap water)	$8.20\cdot10^{-1}$	kg
Heating (market for heat, district or industrial, natural gas)	$1.05\cdot10^{0}$	MJ
Electricity (market for electricity, high voltage)	$7.01\cdot10^{-2}$	kWh

Table S17. Acetic acid synthesis by methanol carbonylation (biogas BAU scenario) LCI.

Functional unit: 1 kg acetic acid		
**Input**	**Amount**	**Units**
Methanol (from biomethane)*	$5.44 \cdot 10^{-1}$	kg
Green carbon monoxide (**Table S8**)	$4.91 \cdot 10^{-1}$	kg
Water (market group for tap water)	$8.20 \cdot 10^{-1}$	kg
Heating (from biomethane) (**Table S11**)	$1.05 \cdot 10^{0}$	MJ
Electricity (market for electricity, high voltage)	$7.01 \cdot 10^{-2}$	kWh

*defined by substituting “Natural gas (market group for natural gas, high pressure)” and “Heating (market for heat, district or industrial, natural gas)” with “Biomethane (market for biomethane, 24 bar w/ CCS)[10]” and “Heating (from biomethane) (**Table S11**)” in the original Ecoinvent v3.8 “Methanol (market for methanol)” activity.

Table S18. Acetic synthesis by methane carboxylation (fossil GTA scenario) LCI.

Functional unit: 1 kg acetic acid		
**Input**	**Amount**	**Units**
Natural gas (market group for natural gas, high pressure)	$4.10 \cdot 10^{-1}$	m^3
Carbon dioxide (from coal power plant capture)[11]	$7.38 \cdot 10^{-1}$	kg
Heating (market for heat, district or industrial, natural gas)	$1.25 \cdot 10^{-2}$	MJ
Cooling (water from 25 to 20 ºC)[9]	$2.19 \cdot 10^{0}$	MJ
Electricity (market for electricity, high voltage)	$7.85 \cdot 10^{-2}$	kWh
**Output**		
Water (emission to air)	$6.78 \cdot 10^{-3}$	m^3
Carbon dioxide (emission to air)	$1.25 \cdot 10^{-2}$	kg

Table S19. Green acetic synthesis by synthetic methane carboxylation (green-DAC GTA scenario) LCI.

Functional unit: 1 kg acetic acid		
**Input**	**Amount**	**Units**
Synthetic methane (from DAC CO_2 and electrolytic H_2) (**Table S6**)	$4.10 \cdot 10^{-1}$	m^3
Carbon dioxide (from DAC) (**Table S14**)	$7.38 \cdot 10^{-1}$	kg
Heating (market for heat, district or industrial, natural gas)	$1.25 \cdot 10^{-2}$	MJ
Cooling (water from 25 to 20 ºC)[9]	$2.19 \cdot 10^{0}$	MJ
Electricity (market for electricity, high voltage)	$7.85 \cdot 10^{-2}$	kWh
**Output**		
Water (emission to air)	$6.78 \cdot 10^{-3}$	m^3
Carbon dioxide (emission to air)	$1.25 \cdot 10^{-2}$	kg

Table S20. Acetic synthesis by biomethane carboxylation (biogas GTA scenario) LCI.

**Functional unit: 1 kg acetic acid**		
**Input**	**Amount**	**Units**
Biogas (market for biogas, 60% biomethane, 40% CO_2)[10]	$6.68 \cdot 10^{-1}$	m^3
Carbon dioxide (from DAC) (**Table S14**)	$2.46 \cdot 10^{-1}$	kg
Heating (market for heat, district or industrial, natural gas)	$1.25 \cdot 10^{-2}$	MJ
Cooling (water from 25 to 20 ºC)[9]	$2.19 \cdot 10^{0}$	MJ
Electricity (market for electricity, high voltage)	$7.85 \cdot 10^{-2}$	kWh
**Output**		
Water (emission to air)	$6.78 \cdot 10^{-3}$	m^3
Carbon dioxide (emission to air)	$1.25 \cdot 10^{-2}$	kg

Table S21. Acetic synthesis by semi-artificial photosynthesis using fossil CO_2 and cysteine as electron donor (fossil SAP scenario) LCI.

Functional unit: 1 kg acetic acid		
**Input**	**Amount**	**Units**
Water (market group for tap water)	$6.00 \cdot 10^{-1}$	kg
Carbon dioxide (from coal power plant capture)[11]	$1.47 \cdot 10^{0}$	kg
Cysteine (from acid hydrolyzation of poultry feathers) (**Table S13**)	$1.60 \cdot 10^{-2}$	kg
Glass tube (glass tube production, borosilicate)*	$8.67 \cdot 10^{-3}$	kg
Aluminium (market for aluminium, cast alloy)**	$2.79 \cdot 10^{-3}$	kg
Electricity (photovoltaic, 570kWp open ground installation, multi-Si)	$5.07 \cdot 10^{0}$	kWh
Battery (market, Li-ion, NMC111, rechargeable, prismatic)	$1.48 \cdot 10^{-2}$	kWh
Cooling (water from 25 to 20 ºC)[9]	$3.29 \cdot 10^{0}$	MJ
Heating (market for heat, district or industrial, natural gas)	$3.45 \cdot 10^{0}$	MJ

*estimated the area obtained from Eq.(S4), 0.04 m of diameter and 0.065 m of thickness[16] and 30 years lifetime of the reactor.

**estimated assuming 0.01 m separation between the reactor tubes and 30 years lifetime of the reactor.

Table S22. Acetic synthesis by semi-artificial photosynthesis using DAC CO_2 and cysteine as electron donor (DAC SAP scenario) LCI.

Functional unit: 1 kg acetic acid		
**Input**	**Amount**	**Units**
Water (market group for tap water)	$6.00\cdot10^{-1}$	kg
Carbon dioxide (from DAC) (**Table S14**)	$1.47\cdot10^{0}$	kg
Cysteine (from acid hydrolyzation of poultry feathers) (**Table S13**)	$1.60\cdot10^{-2}$	kg
Glass tube (glass tube production, borosilicate)*	$8.67\cdot10^{-3}$	kg
Aluminium (market for aluminium, cast alloy)**	$2.79\cdot10^{-3}$	kg
Electricity (photovoltaic, 570kWp open ground installation, multi-Si)	$5.07\cdot10^{0}$	kWh
Battery (market, Li-ion, NMC111, rechargeable, prismatic)	$1.48\cdot10^{-2}$	kWh
Cooling (water from 25 to 20 ºC)[9]	$3.29\cdot10^{0}$	MJ
Heating (market for heat, district or industrial, natural gas)	$3.45\cdot10^{0}$	MJ

*estimated the area obtained from Eq.(S4), 0.04 m of diameter and 0.065 m of thickness[16] and 30 years lifetime of the reactor.

**estimated assuming 0.01 m separation between the reactor tubes and 30 years lifetime of the reactor.

Table S23. Acetic synthesis by semi-artificial photosynthesis using fossil CO_2 and water as electron donor (fossil SAP with water scenario) LCI.

**Functional unit: 1 kg acetic acid**		
**Input**	**Amount**	**Units**
Water (market group for tap water)	$6.00 \cdot 10^{-1}$	kg
Carbon dioxide (from coal power plant capture)[11]	$1.47 \cdot 10^{0}$	kg
Glass tube (glass tube production, borosilicate)*	$8.67 \cdot 10^{-3}$	kg
Aluminium (market for aluminium, cast alloy)**	$2.79 \cdot 10^{-3}$	kg
Electricity (photovoltaic, 570kWp open ground installation, multi-Si)	$6.10 \cdot 10^{-1}$	kWh
Battery (market, Li-ion, NMC111, rechargeable, prismatic)	$1.78 \cdot 10^{-3}$	kWh
Cooling (water from 20 to 25 ºC)[9]	$3.29 \cdot 10^{0}$	MJ
Heating (market for heat, district or industrial, natural gas)	$3.45 \cdot 10^{0}$	MJ

*estimated the area obtained from Eq.(S4), 0.04 m of diameter and 0.065 m of thickness[16] and 30 years lifetime of the reactor.

**estimated assuming 0.01 m separation between the reactor tubes and 30 years lifetime of the reactor.

Table S24. Acetic synthesis by semi-artificial photosynthesis using DAC CO_2 and water as electron donor (DAC SAP with water scenario) LCI.

**Functional unit: 1 kg acetic acid**		
**Input**	**Amount**	**Units**
Water (market group for tap water)	$6.00 \cdot 10^{-1}$	kg
Carbon dioxide (from DAC) (**Table S14**)	$1.47 \cdot 10^{0}$	kg
Glass tube (glass tube production, borosilicate)*	$8.67 \cdot 10^{-3}$	kg
Aluminium (market for aluminium, cast alloy)**	$2.79 \cdot 10^{-3}$	kg
Electricity (photovoltaic, 570kWp open ground installation, multi-Si)	$6.10 \cdot 10^{-1}$	kWh
Battery (market, Li-ion, NMC111, rechargeable, prismatic)	$1.78 \cdot 10^{-3}$	kWh
Cooling (water from 25 to 20 ºC)[9]	$3.29 \cdot 10^{0}$	MJ
Heating (market for heat, district or industrial, natural gas)	$3.45 \cdot 10^{0}$	MJ

*estimated the area obtained from Eq.(S4), 0.04 m of diameter and 0.065 m of thickness[16] and 30 years lifetime of the reactor.

**estimated assuming 0.01 m separation between the reactor tubes and 30 years lifetime of the reactor.

B.2. Monte Carlo analysis results

In this section, we present the complementary results of the endpoint uncertainty analyses for the assessed technologies and compare them with the three BAU variants (fossil, green-DAC and biogas).

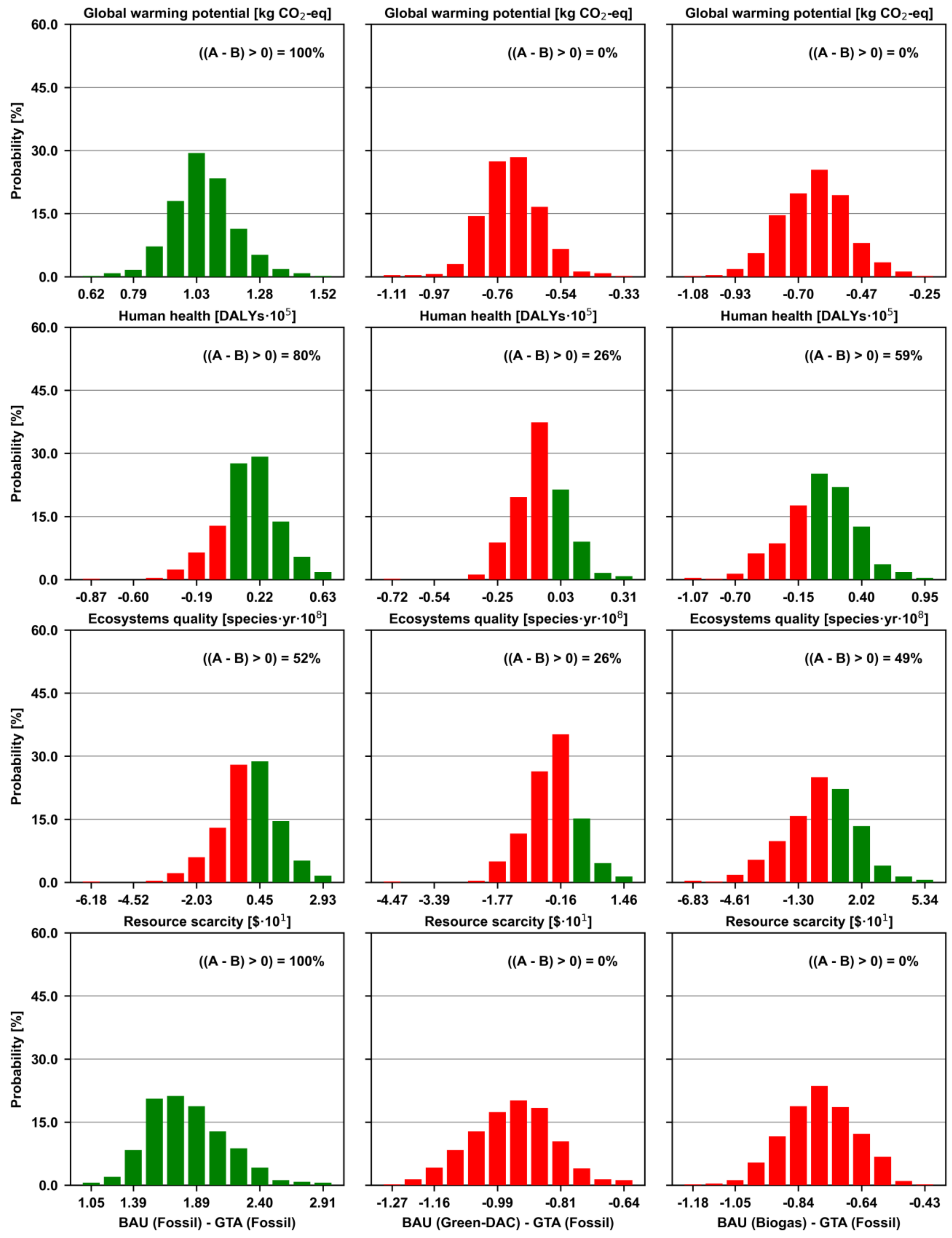

Figure S6. Uncertainty analysis of the three BAU (Fossil, Green-DAC and Biogas) scenarios (A) minus the GTA (Fossil) scenario (B). A result lower than zero is indicative of burden shifting.

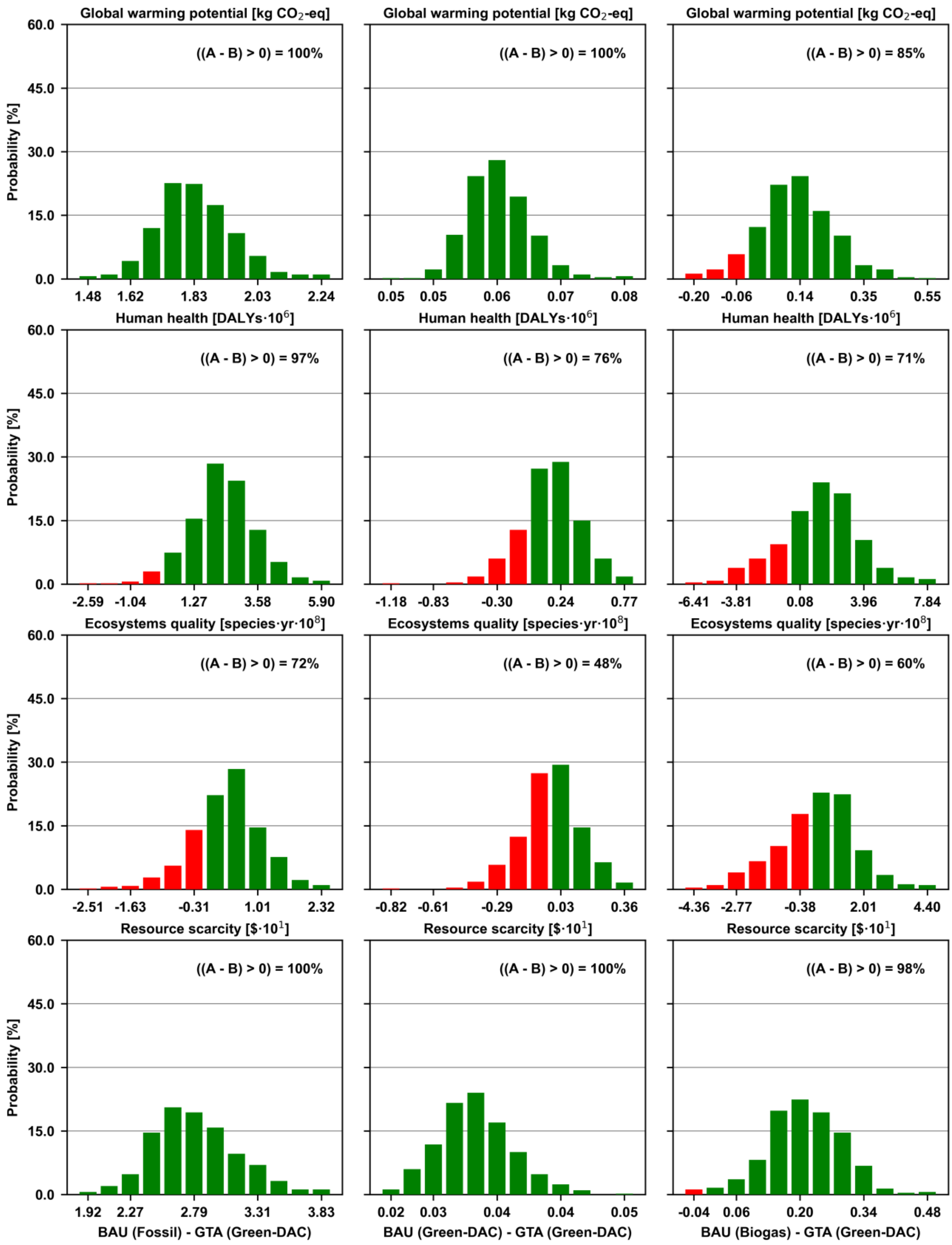

Figure S7. Uncertainty analysis of the three BAU (Fossil, Green-DAC and Biogas) scenarios (A) minus the GTA (green-DAC) scenario (B). A result lower than zero is indicative of burden shifting.

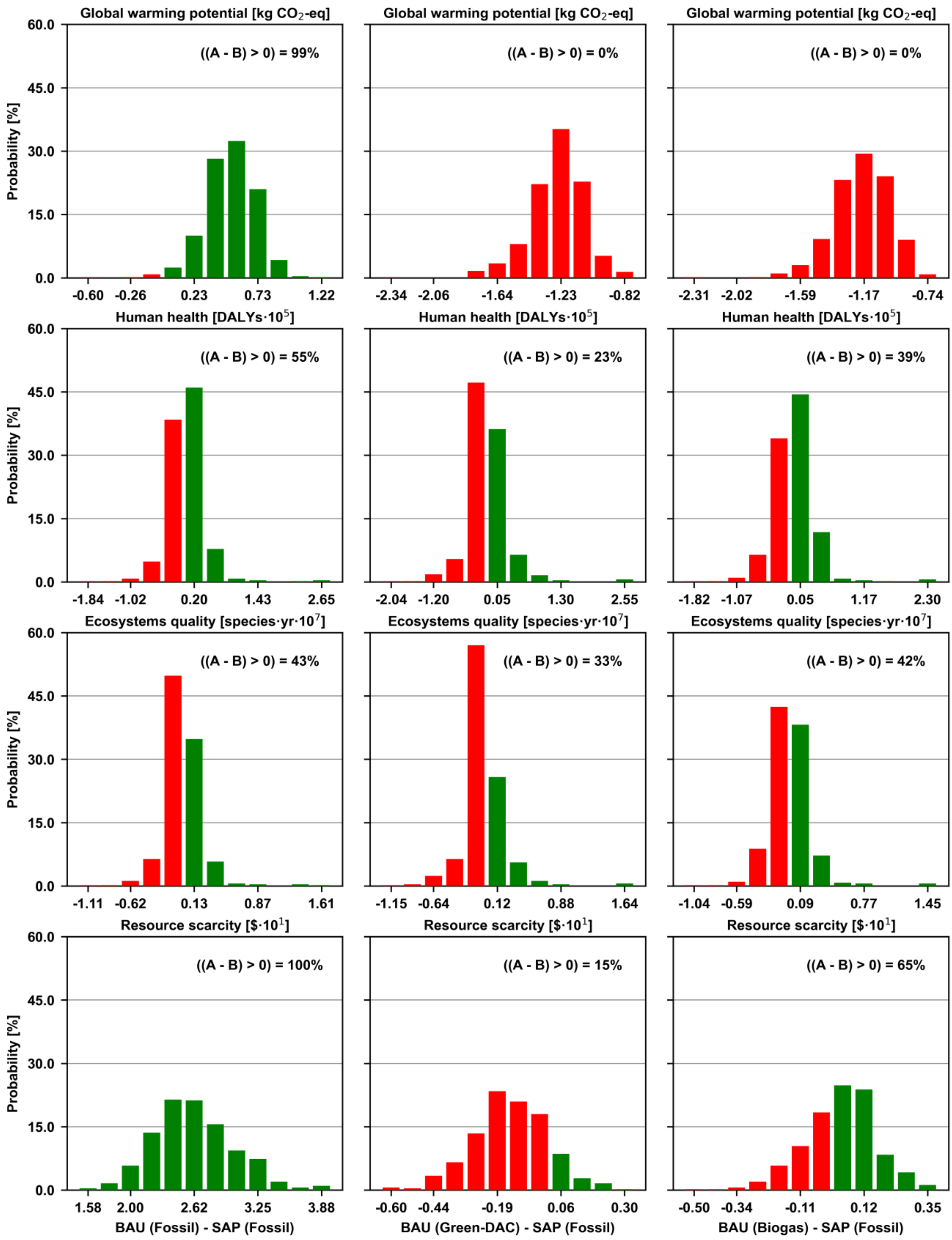

Figure S8. Uncertainty analysis of the three BAU (Fossil, Green-DAC and Biogas) scenarios (A) minus the SAP (Fossil) scenario (B). A result lower than zero is indicative of burden shifting.

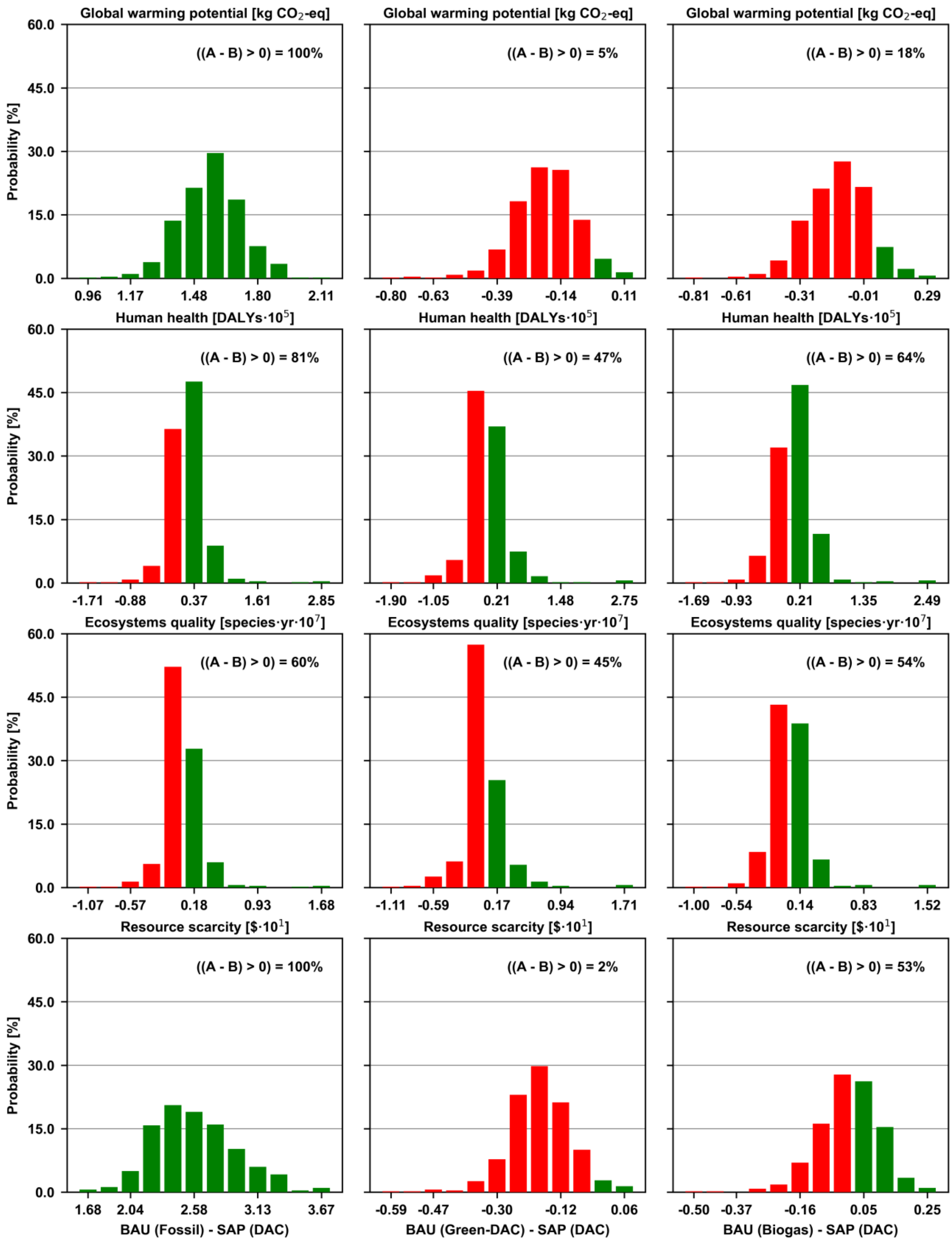

Figure S9. Uncertainty analysis of the three BAU (Fossil, Green-DAC and Biogas) scenarios (A) minus the SAP (DAC) scenario (B). A result lower than zero is indicative of burden shifting.

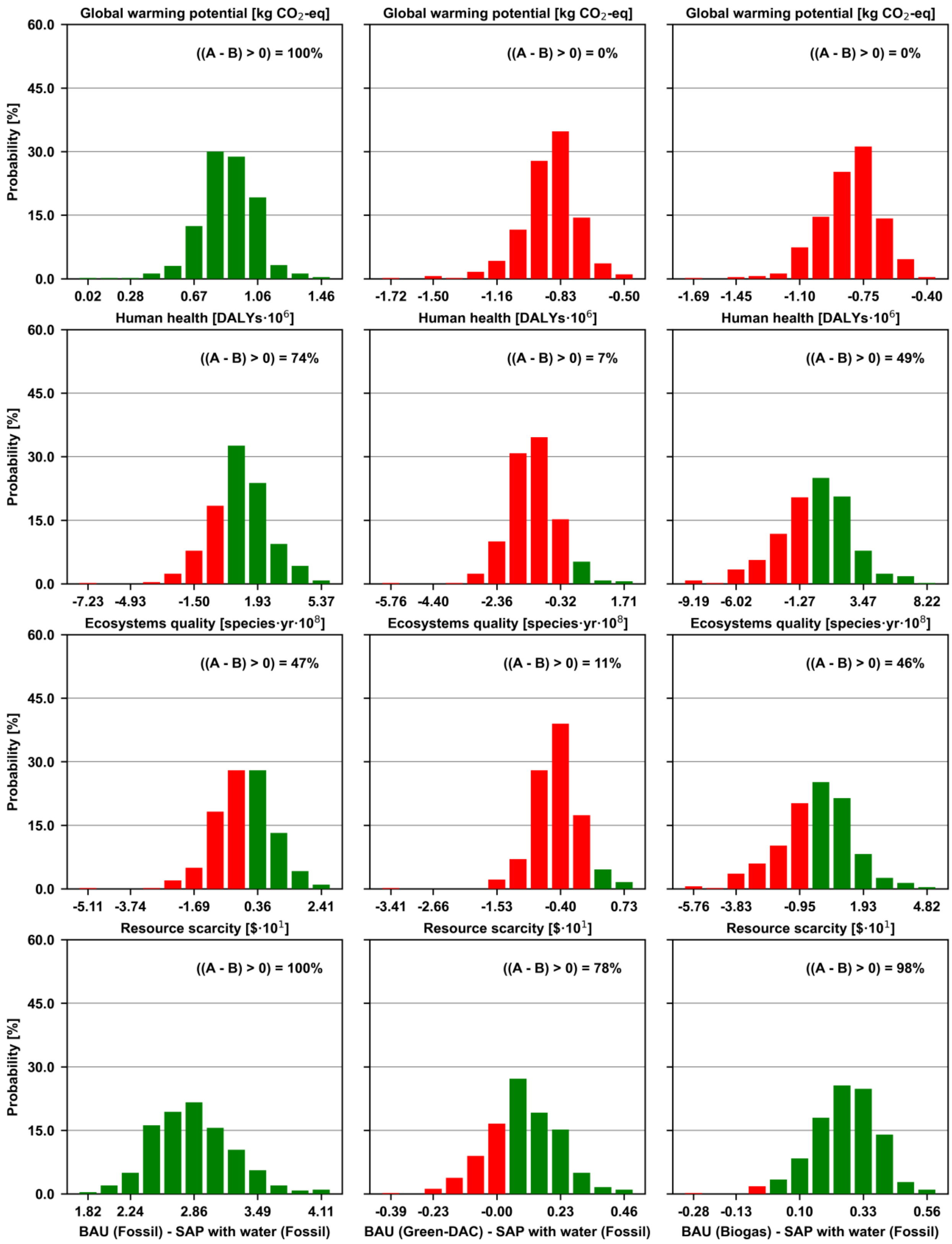

Figure S10. Uncertainty analysis of the three BAU (Fossil, Green-DAC and Biogas) scenarios (A) minus the SAP with water (Fossil) scenario (B). A result lower than zero is indicative of burden shifting.

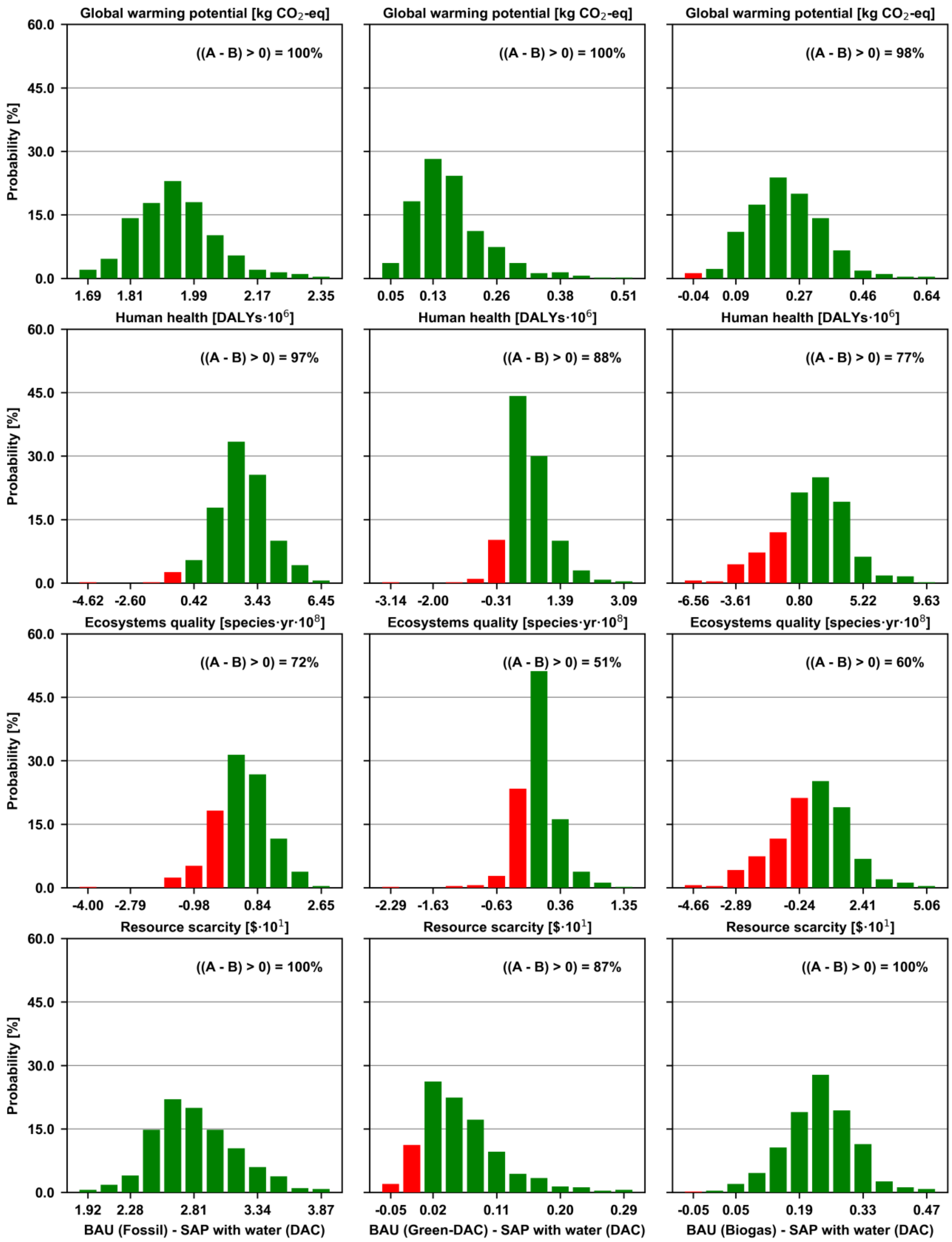

Figure S11. Uncertainty analysis of the three BAU (Fossil, Green-DAC and Biogas) scenarios (A) minus the SAP with water (DAC) scenario (B). A result lower than zero is indicative of burden shifting.

C. Economic assessment

In this section, we provide the data employed in the economic study. First, we show the costs of required materials and energy and then we describe the capital cost calculation.

C.1. Raw materials and utility costs

Table S25. Base material and utility prices.

Input	Amount	Units	Reference
Methane (from natural gas)	$2.85 \cdot 10^{-1}$	$\$ \cdot m^{-3}$	[17]
Biogas	$1.83 \cdot 10^{-1}$	$\$ \cdot m^{-3}$	[18]
Biomethane	$7.00 \cdot 10^{-1}$	$\$ \cdot m^{-3}$	[19]
Methanol (fossil)	$4.50 \cdot 10^{-1}$	$\$ \cdot kg^{-1}$	[20]
O_2 (from air separation)	$1.23 \cdot 10^{-1}$	$\$ \cdot kg^{-1}$	[9]
H_2 (from wind electricity)	$5.24 \cdot 10^{0}$	$\$ \cdot kg^{-1}$	[21]
CO_2 (from DAC)	$2.00 \cdot 10^{-1}$	$\$ \cdot kg^{-1}$	[11]
CO_2 (from coal power plant capture)	$3.95 \cdot 10^{-2}$	$\$ \cdot kg^{-1}$	[11]
Cysteine	$6.00 \cdot 10^{2}$	$\$ \cdot kg^{-1}$	[13]
Borosilicate tube	$5.00 \cdot 10^{-0}$	$\$ \cdot kg^{-1}$	[22]
Aluminum	$2.20 \cdot 10^{0}$	$\$ \cdot kg^{-1}$	[23]
Water	$1.77 \cdot 10^{-4}$	$\$ \cdot kg^{-1}$	[17]
Heating (from natural gas)	$3.51 \cdot 10^{-3}$	$\$ \cdot MJ^{-1}$	[17]
Cooling (water from 25 to 20 ºC)	$3.78 \cdot 10^{-4}$	$\$ \cdot MJ^{-1}$	[17]
Electricity (grid)	$1.10 \cdot 10^{-1}$	$\$ \cdot kWh^{-1}$	[18]
Electricity (solar)*	$4.00 \cdot 10^{-2}$	$\$ \cdot kWh^{-1}$	[24]
Battery (Li-ion)	$2.50 \cdot 10^{1}$	$\$ \cdot kg^{-1}$	[16,17]

*in the range of the global average (0.044 kWh) and Spanish (0.038 $/kWh) reported values

Table S26. Calculated material and utility prices.

Input	Amount	Units	LCI
Green methanol	$1.34 \cdot 10^{-0}$	$\$ \cdot kg^{-1}$	[15]
Methanol (from biomethane)	$4.89 \cdot 10^{-1}$	$\$kg^{-1}$	[25]
CO (from natural gas POX)	$4.00 \cdot 10^{-1}$	$\$ \cdot kg^{-1}$	**Table S7**
Green CO (from CO_2 and H_2)	$7.08 \cdot 10^{-1}$	$\$ \cdot kg^{-1}$	**Table S9**
CO (from biomethane POX)	$5.98 \cdot 10^{-1}$	$\$ \cdot kg^{-1}$	**Table S8**
Synthetic methane (from the Sabatier reaction)	$3.30 \cdot 10^{0}$	$\$ \cdot kg^{-1}$	**Table S12**

C.2. Capital investment cost

The fixed capital cost (total fixed capital cost, *CAPEX*) is based on Sinnott and Towler[26]. First, we estimate the purchased equipment costs from the capacity using Eq.(S10):

$$C_{e,i} = a_i + b_i S_i^{n_i} \tag{S10}$$

Where $C_{e,i}$ is the purchased equipment cost of i in 2010 US $, a_i, b_i and n_i are constants, and S_i is the size parameter measured in different units depending on the type of equipment i. These data can be found in Sinnot and Towler[26]. Note that the cost of the photosynthesis reactor is instead calculated directly from the material cost, while both the cysteine regeneration electrolyzer and the electrodialysis unit are considered at 800 $/kW[11].

Then, we update these costs from 2010 to 2019 using the Chemical Engineering Plant Cost Index (CEPCI) (Eq.(S11)):

$$\text{Cost in year A} = \text{Cost in year B}\frac{\text{Cost index in year A}}{\text{Cost index in year B}} \tag{S11}$$

Where Cost in year B and Cost in year A are the purchased equipment costs [$] for the years 2010 and 2019 and $\text{Cost index in year B}$ (532.9) and $\text{Cost index in year A}$ (607.5) are the CEPCI annual mean values for the corresponding years.

With the updated purchased equipment cost, we add the factors for equipment erection (f_{er} = 0.3), piping (f_p = 0.8) instrumentation and process control (f_i = 0.3), electrical work (f_{el} = 0.2), civil engineering work (f_c = 0.3), structures and buildings (f_s = 0.2), lagging, insulation or paint (f_l = 0.1) and material factor (f_m = 1.0 for carbon steel) to calculate the installed capital cost (C , $) using Eq.(S12):

$$C = \sum_i C_{e,i}\left[(1+f_p) + \frac{f_{er}+f_{el}+f_i+f_c+f_s+f_l}{f_m}\right] \tag{S12}$$

Then, we add the offsites (*OS* = 0.3), design and engineering (*D&E* = 0.3), and contingency (*X* = 0.1) to get the *CAPEX* [$] (Eq.(S13)):

$$CAPEX = C\left(1+OS\right)\left(1+D\&E+X\right) \tag{S13}$$

We calculate the annualization factor (or annual capital charge ratio, *ACCR*) considering a 30-year lifetime of the plant and an interest rate of 0.1 as described in Eq.(S14):

$$ACCR = \frac{\left[i\left(1+i\right)^{n}\right]}{\left[\left(i+1\right)^{n}-1\right]} \tag{S14}$$

Finally, the product of the *ACCR* and the *CAPEX* results in the annualized capital investment (*ACC*, $/y) (Eq.(S15)):

$$ACC = CAPEX \cdot ACCR \tag{S15}$$

D. Assumptions

In this section, we present the list of the most important assumptions made in this work for the simulations, environmental and economic assessments. Furthermore, we show the minum and maximum price ranges for the economic sensitivity analysis.

D.1. Process design assumptions

- Equilibrium Gibbs reactors are assumed for fossil and green CO synthesis and green synthetic methane production (best-case scenario). However, the scenarios where these technologies are used (fossil BAU, green BAU and green GTA) are outperformed by the biogas GTA and the DAC SAP using water. Hence, we conclude that using a kinetic model in said reactors would only decrease the performance even more, thus still favoring the biogas GTA and and the DAC SAP using water.
- The catalytic experimental performance (conversion, selectivity and efficiencies) for the GTA and SAP systems was assumed to scale linearly to the industrial scale.
- The photosynthesis medium loss in the SAP simulations was assumed to be 0.1%, and the medium composition was only water.
- Cystine was assumed to be separated by precipitation.
- Cysteine was assumed to be regenerated electrolytically at a 50% energy efficiency, with a 0.1% mass loss.
- Acetic acid was assumed to be concentrated by electrodialysis with a linear electricity consumption based on the mass of acetic acid and the final purity achieved.
- Water was assumed to substitute cysteine without any major change in the process performance (conversion, selectivity or efficiency).
- For CAPEX calculations, all scenarios were assumed to scale to the BAU production capacity.

D.2. Economic sensitivity analysis data

Table S27. Raw material price range based on frequently cited literature. Biogas was based on the European Biogas Association reports, while biomethane was estimated from the biogas price.

Item	Raw material	Min	Max	Units	Reference
1	Biogas	$6.60\cdot10^{-2}$	$3.00\cdot10^{-1}$	$\$\cdot m^{-3}$	[18]
2	Biomethane	$2.52\cdot10^{-1}$	$1.15\cdot10^{0}$	$\$\cdot m^{-3}$	-
3	Methane (natural gas)	$8.47\cdot10^{-2}$	$4.37\cdot10^{-1}$	$\$\cdot m^{-3}$	[17]
4	Methanol (fossil)	$3.80\cdot10^{-1}$	$6.95\cdot10^{-1}$	$\$\cdot kg^{-1}$	[20]
5	H_2 (green)	$3.56\cdot10^{0}$	$1.08\cdot10^{1}$	$\$\cdot kg^{-1}$	[21]
6	CO_2 (fossil)	$3.30\cdot10^{-2}$	$5.80\cdot10^{-2}$	$\$\cdot kg^{-1}$	[27]
7	CO_2 (DAC)	$9.40\cdot10^{-2}$	$2.32\cdot10^{-1}$	$\$\cdot kg^{-1}$	[14]
8	Methane (green)	$2.09\cdot10^{0}$	$6.20\cdot10^{0}$	$\$\cdot m^{-3}$	-
9	Methanol (green)	$8.27\cdot10^{-1}$	$2.43\cdot10^{0}$	$\$\cdot kg^{-1}$	-
10	Methanol (bio)	$3.59\cdot10^{-1}$	$1.26\cdot10^{0}$	$\$\cdot kg^{-1}$	-
11	CO (fossil)	$2.17\cdot10^{0}$	$5.40\cdot10^{0}$	$\$\cdot kg^{-1}$	-
12	CO (green)	$8.28\cdot10^{-1}$	$2.44\cdot10^{0}$	$\$\cdot kg^{-1}$	-
13	CO (bio)	$5.68\cdot10^{-1}$	$1.39\cdot10^{0}$	$\$\cdot kg^{-1}$	-

1. Assumed 60% volume of methane in biogas.

2. Assumed the same variance of biogas prices with 0.7 $\$/m^3$ as the base

3. August 2024 natural gas US price (minimum) and Europe price (maximum)

4. August 2024 China price (minimum) and North America price (maximum)

5. Wind electrolysis

6. Post-combustion coal power plant

8-13. Calculated from their respective inventories.

E. References

(1) Carlson, E. C. Don't Gamble With Physical Properties For Simulations. *Chemical Engineering Progress*. 1996, pp 35–46.

(2) Medrano-García, J. D.; Ruiz-Femenia, R.; Caballero, J. A. Optimal Carbon Dioxide and Hydrogen Utilization in Carbon Monoxide Production. *J. CO2 Util.* **2019**, *34*, 215–230. https://doi.org/10.1016/j.jcou.2019.05.005.

(3) Gai, P.; Yu, W.; Zhao, H.; Qi, R.; Li, F.; Liu, L.; Lv, F.; Wang, S. Solar-Powered Organic Semiconductor–Bacteria Biohybrids for CO_2 Reduction into Acetic Acid. *Angew. Chemie - Int. Ed.* **2020**, *59* (18), 7224–7229. https://doi.org/10.1002/anie.202001047.

(4) Yu, L.; Guo, Q.; Hao, J.; Jiang, W. Recovery of Acetic Acid from Dilute Wastewater by Means of Bipolar Membrane Electrodialysis. *Desalination* **2000**, *129* (3), 283–288. https://doi.org/10.1016/S0011-9164(00)00068-0.

(5) Zhang, X.; Li, C.; Wang, Y.; Luo, J.; Xu, T. Recovery of Acetic Acid from Simulated Acetaldehyde Wastewaters: Bipolar Membrane Electrodialysis Processes and Membrane Selection. *J. Memb. Sci.* **2011**, *379* (1–2), 184–190. https://doi.org/10.1016/j.memsci.2011.05.059.

(6) Moreno-Tejera, S.; Silva-Pérez, M. A.; Lillo-Bravo, I.; Ramírez-Santigosa, L. Solar Resource Assessment in Seville, Spain. Statistical Characterisation of Solar Radiation at Different Time Resolutions. *Sol. Energy* **2016**, *132*, 430–441. https://doi.org/10.1016/j.solener.2016.03.032.

(7) Sen, C. K.; Packer, L. Thiol Homeostasis and Supplements in Physical Exercise. *Am. J. Clin. Nutr.* **2000**, *72* (2 SUPPL.). https://doi.org/10.1093/ajcn/72.2.653s.

(8) Sakimoto, K. K.; Wong, A. B.; Yang, P. Self-Photosensitization of Nonphotosynthetic

Bacteria for Solar-to-Chemical Production. *Science (80-.).* **2016**, *351* (6268), 74–77. https://doi.org/10.1126/science.aad3317.

(9) Medrano-García, J. D.; Giulimondi, V.; Ceruti, A.; Zichittella, G.; Pérez-Ramírez, J.; Guillén-Gosálbez, G. Economic and Environmental Competitiveness of Ethane-Based Technologies for Vinyl Chloride Synthesis. *ACS Sustain. Chem. Eng.* **2023**, *11* (35), 13062–13069. https://doi.org/10.1021/acssuschemeng.3c03006.

(10) Istrate, R. Dataset and Code: One-Tenth of EU's Biomethane Potential Combined with Carbon Capture and Storage Can Shift the Region's Ammonia Production to Net- Zero. Zenodo October 2024. https://doi.org/10.5281/zenodo.13907125.

(11) Medrano-García, J. D.; Charalambous, M. A.; Guillén-Gosálbez, G. Economic and Environmental Barriers of CO2-Based Fischer-Tropsch Electro-Diesel. *ACS Sustain. Chem. Eng.* **2022**, *10* (36), 11751–11759. https://doi.org/10.1021/acssuschemeng.2c01983.

(12) Luyben, W. L. Estimating Refrigeration Costs at Cryogenic Temperatures. *Comput. Chem. Eng.* **2017**, *103*, 144–150. https://doi.org/10.1016/j.compchemeng.2017.03.013.

(13) Shian, C. Method for Producing L-Cystine. CN102887844A.

(14) Keith, D. W.; Holmes, G.; St. Angelo, D.; Heidel, K. A Process for Capturing CO2 from the Atmosphere. *Joule* **2018**, *2* (8), 1573–1594. https://doi.org/10.1016/j.joule.2018.05.006.

(15) González-Garay, A.; Frei, M. S.; Al-Qahtani, A.; Mondelli, C.; Guillén-Gosálbez, G.; Pérez-Ramírez, J. Plant-to-Planet Analysis of CO 2-Based Methanol Processes. *Energy Environ. Sci.* **2019**, *12* (12), 3425–3436.

(16) Zacarías, S. M.; Manassero, A.; Pirola, S.; Alfano, O. M.; Satuf, M. L. Design and

Performance Evaluation of a Photocatalytic Reactor for Indoor Air Disinfection. *Environ. Sci. Pollut. Res.* **2021**, *28* (19), 23859–23867. https://doi.org/10.1007/s11356-020-11663-6.

(17) World Bank Group. *Commodity markets*. https://www.worldbank.org/en/research/commodity-markets (accessed 2024-07-25).

(18) European Biogas Association (EBA). *IRENA publication: Biogas cost reductions to boost sustainable transport*. https://www.europeanbiogas.eu/irena-publication-biogas-cost-reductions-boost-sustainable-transport/ (accessed 2024-07-25).

(19) Wouters, C.; Buseman, M.; Tilburg, J. van; Berg, T.; Cihlar, J.; Ainhoa Villar Lejarreta, J. J.; Wang, A.; Peters, D.; Leun, K. van der. *Market State and Trends in Renewable and Low-Carbon Gases in Europe*; 2020. https://www.consorziobiogas.it/wp-content/uploads/2020/02/Gas-for-Climate-Market-State-and-Trends-report-2020.pdf.

(20) Methanex Corporation. *Methanex Methanol Price Sheet*. Pricing. https://www.methanex.com/about-methanol/pricing/.

(21) Parkinson, B.; Balcombe, P.; Speirs, J. F.; Hawkes, A. D.; Hellgardt, K. Levelized Cost of CO2 Mitigation from Hydrogen Production Routes. *Energy Environ. Sci.* **2019**, *12* (1), 19–40. https://doi.org/10.1039/c8ee02079e.

(22) Alibaba.com. *Heat resistance borosilicate glass tube*. https://www.alibaba.com/product-detail/Heat-Resistance-Borosilicate-Glass-Tube-Clear_62478871294.html?spm=a2700.7724857.0.0.23645a0cD5VaCb (accessed 2024-07-25).

(23) Trading economics. *Aluminum*. https://tradingeconomics.com/commodity/aluminum (accessed 2024-07-25).

(24) IRENA. *Renewable Power Generation Costs in 2023*; 2024. https://www.irena.org/Publications/2024/Sep/Renewable-Power-Generation-Costs-in-2023.

(25) Wernet, G.; Bauer, C.; Steubing, B.; Reinhard, J.; Moreno-Ruiz, E.; Weidema, B. The Ecoinvent Database Version 3 (Part I): Overview and Methodology. *Int. J. Life Cycle Assess.* **2016**, *21* (9), 1218–1230. https://doi.org/10.1007/s11367-016-1087-8.

(26) Towler, G.; Sinnott, R. K. *Chemical Engineering Design*; Elsevier Ltd, 2013. https://doi.org/10.1016/C2009-0-61216-2.

(27) Rubin, E. S.; Davison, J. E.; Herzog, H. J. The Cost of CO2 Capture and Storage. *Int. J. Greenh. Gas Control* **2015**, *40*, 378–400. https://doi.org/10.1016/j.ijggc.2015.05.018.
